# The diagnostic performance of cochlear endolymphatic hydrops and perilymphatic enhancement in stratifying Ménière’s disease probabilities: A meta-analysis of semi-quantitative MRI-based grading systems

**DOI:** 10.1371/journal.pone.0310045

**Published:** 2024-11-21

**Authors:** Neda Azarpey, Shahrzad-Sadat Seyed-Bagher-Nazeri, Omid Yazdani, Romina Esbati, Paria Boustani, Mobasher Hajiabbasi, Pouya Torabi, Dorreh Farazandeh, Hana Farzaneh, Ashkan Azizi, Behnam Amini, Moein Ghasemi, Zohre Ghasemi

**Affiliations:** 1 Department of Radiology, Shahid Beheshti University, Tehran, Iran; 2 Faculty of Medicine, Tehran University of Medical Sciences, Tehran, Iran; 3 Faculty of Medicine, Islamic Azad University of Tonekabon, Tonekabon, Iran; 4 Department of Radiology, Iran University of Medical Sciences, Tehran, Iran; 5 Department of Otorhinolaryngology-Head and Neck Surgery, Imam Khomeini Hospital Complex, Tehran University of Medical Sciences, Tehran, Iran; University of Porto Faculty of Medicine, PORTUGAL

## Abstract

**Background:**

The diagnosis of Meniere’s Disease (MD) presents significant challenges due to its complex symptomatology and the absence of definitive biomarkers. Advancements in MRI technology have spotlighted endolymphatic hydrops (EH) as a key pathological marker, necessitating a reevaluation of its diagnostic utility amidst the need for standardized and validated MRI-based grading scales.

**Methods:**

Our meta-analysis scrutinized the diagnostic efficacy of semi-quantitative MRI-based cochlear endolymphatic hydrops (EH) and perilymphatic enhancement (PLE) grading systems in delineating clinically relevant discriminations: “Spotting” the shift from normal or asymptomatic ears to possible/probable MD (pMD), “Confirming” the progression to definite MD (dMD), and “Establishing” the presence of dMD. A thorough literature search up to October 2023 resulted in 35 pertinent studies, forming the basis of our analysis through a bivariate mixed-effects regression model.

**Results:**

Using criteria from the American Academy of Otolaryngology-Head and Neck Surgery (AAO-HNS) and Barany Society, across varying thresholds and disease probabilities; the Establishment model at an EH grade 1 threshold revealed a sensitivity of 85.4% and a specificity of 82.7%. Adjusting the threshold to EH grade 2 results in a sensitivity increase to 92.1% (CI: 85.9–95.7) and a specificity decrease to 70.6% (CI: 64.5–76.1), with a DOR of 28.056 (CI: 14.917–52.770). The Confirmation model yields a DOR of 5.216, indicating a lower diagnostic accuracy. The Spotting model demonstrates a sensitivity of 48.3% (CI: 34.8–62.1) and a specificity of 88.0% (CI: 77.8–93.9), with a DOR of 6.882. The normal ears subgroup demonstrated a notably high specificity of 89.7%, while employing Nakashima’s criteria resulted in a reduced sensitivity of 74.9%, significantly diverging from other systems (p-value < 0.001). The PLE grading system showcased exceptional sensitivity of 98.4% (CI: 93.7–99.6, p-value < 0.001).

**Conclusion:**

Our meta-analysis supports a tailored diagnostic approach for MD, emphasizing the need for effective grading systems at each stage. For "Spotting," the model shows high specificity but requires improved sensitivity, suggesting additional criteria are needed. The "Confirming" stage highlights the need for refined, sensitive grading systems due to lower diagnostic accuracy. In the "Establishing" stage, an EH grade 1 threshold is effective, but grade 2 enhances sensitivity while reducing specificity, indicating a need for balance. The PLE grading system excels in sensitivity, making it highly reliable. High specificity in the normal ears subgroup confirms accurate non-pathological distinction, though Nakashima’s criteria show reduced sensitivity, underscoring variability in grading systems. These findings advocate for a standardized, unified grading system balancing sensitivity and specificity across all MD stages to optimize diagnostics and clinical outcomes.

## Introduction

Ménière’s Disease (MD) encapsulates a significant challenge within the otolaryngology and neurology, characterized by its indeterminate symptomatology and the absence of concrete biomarkers for diagnosis [1, 2]. The reliance on clinical criteria and patient-reported symptoms further complicates the diagnostic process, especially in the disease’s nascent stages [3, 4]. The unclear nature of MD’s etiology remains a topic of significant interest, with endolymphatic hydrops (EH) posited as a pivotal factor in its pathogenesis, characterized by an aberrant fluid accumulation within the inner ear’s endolymphatic spaces [3–5]. This association has catalyzed advancements in diagnostic methodologies, notably through delayed post-gadolinium MRI techniques, closing the gap between subjective clinical assessments and objective diagnostic indicators [3, 4, 6].

The introduction of gadolinium contrast in MRI scans has been a pivotal development for MD diagnostics, enabling the demarcation of endolymphatic spaces as contrast defects and facilitating a correlation between the degree of EH and the clinical manifestations of MD [3, 4, 6]. Despite this progress, the diagnostics of MD is hindered by the variability of grading systems, ranging from qualitative to semi-quantitative and volumetric scales. This diagnostic conundrum is further exacerbated by the incidental discovery of EH in asymptomatic individuals through MRI, obscuring distinctions between disease severity and physiological variance [3, 4, 6].

The development and implementation of standardized grading scales for MRI-based diagnostics of EH in MD patients remain a formidable challenge. The inconsistency in grading systems underscores a significant hurdle in achieving consensus on diagnostic criteria, complicating the interpretation and comparability of research findings [6]. The literature has seen various attempts [7–12] to consolidate MRI-based evaluations of EH in MD, with Connor et al. [12]’s meta-analysis standing out as a seminal work that navigated through the complexities of diagnostic performance with a quantitative lens. However, the study’s approach to amalgamating patient-based and ear-based EH measurements, the unilateral application of the Barany criteria as the definitive standard, and the inclusion of ears with alternate audio-vestibular disorders in the control group, introduces potential biases that may skew the interpretation of EH’s role in MD.

Our study seeks to transcend these limitations through a methodologically robust approach, prioritizing categorization of disease probabilities, stringent selection criteria, and a discerning adoption of diagnostic standards. Our analysis delves into the diagnostic veracity of various EH grading systems, aiming to “Spotting” the shift from normal or asymptomatic ears to probable MD (pMD), “Confirming” the progression to definite MD (dMD), and “Establishing” the presence of dMD. By evaluating the efficacy of semi-quantitative grading systems for cochlear EH and perilymphatic enhancement (PLE), our study provides new insights on the diagnostic utility of these scales in refining the diagnostic approach for MD [1, 2].

## Methods and materials

### Search strategy

A comprehensive literature search was conducted across PubMed, Scopus, Web of Science, and the Cochrane Library to identify studies relevant to the diagnostic accuracy of endolymphatic hydrops (EH) grading in Meniere’s disease. This search, crafted in collaboration with a medical librarian, was peer-reviewed using the Peer Review of Electronic Search Strategies (PRESS) guidelines. The strategy combined keywords and Medical Subject Headings (MeSH) terms, including "Meniere", "cochlear Disease", "endolymphatic hydrops", "Imaging", "Magnetic Resonance", "MRI", "FLAIR", and "Three-Dimensional Imaging", using Boolean operators to ensure breadth and depth. The search was limited to articles published in English up to October 10, 2023, to capture the most current research within the constraints of available translation resources. In selecting studies, a phased approach was employed, focusing on reports detailing diagnostic metrics of cochlear EH grading, as well as perilymphatic enhancement (PLE) evaluation in Meniere’s disease. The PICOS framework guided the selection, encompassing participants diagnosed with MD per established criteria, MRI acquisition methodologies, control groups, outcomes related to EH visualization, and study designs including randomized case-control, prospective and retrospective cohort, and cross-sectional studies. Exclusions were made for reviews, editorials, insufficient data, and animal studies, along with studies with non-discriminatory EH reporting or focusing solely on audio-vestibular symptoms in non-control ears. The screening process began with titles and abstracts, advancing eligible studies to full-text review in adherence to PRISMA-Diagnostic Test Accuracy (PRISMA-DTA) guidelines [13]. This process was meticulously documented in a PRISMA flow diagram. Two independent reviewers (N.A./B.A.) conducted the screenings. Disagreements were resolved through discussion or consultation with a third reviewer in medical statistics, ensuring a thorough and unbiased study selection.

### Data extraction

Data extraction for this study was carried out using a pre-designed Excel spreadsheet. The parameters included first author, publication year, patient inclusion criteria, sample size, methods of gadolinium-based contrast administration (either intratympanic or intravenous), levels of Meniere’s disease diagnostic certainty, locations of endolymphatic hydrops (EH), the status of control ears, and diagnostic test results. The extraction process involved manual entry by two independent reviewers into a shared database, minimizing transcription errors. Discrepancies encountered during data extraction were resolved through a reconciliation process, which included revisiting the original articles and, if necessary, consulting a third reviewer.

### Quality assessment

The quality of the included studies was rigorously assessed using the Quality Assessment of Diagnostic Accuracy Studies-2 (QUADAS-2) tool [14]. This tool provides an in-depth evaluation across four domains: patient selection, index test, reference standard, and flow and timing. Each domain was evaluated independently by the reviewers, with the risk of bias rated as ’low’, ’high’, or ’unclear’. These ratings were based on predefined criteria aligned with QUADAS-2 guidelines. Additionally, the first three domains were also appraised for their relevance and applicability to the research question.

### Data synthesis and analysis

A bivariate mixed-effects regression model was utilized to jointly model sensitivity and specificity, taking into account the heterogeneity observed between studies. The criteria for pooling studies in the meta-analysis were stringently defined, requiring at least three studies examining the same index test and diagnosis and congruence in population characteristics, test applications, and methodologies, as assessed through clinical judgment. To provide a comprehensive assessment of heterogeneity, various statistical measures were employed. These included the variances of logit-transformed sensitivity and specificity, bivariate I^2^, and the area of the 95% prediction ellipse. We also involved subgroup analyses and meta-regression to explore potential sources of variability among the studies. All statistical analyses were carried out using R (version 4.3.0; R Foundation for Statistical Computing, Vienna, Austria), incorporating specialized packages such as lmtest for likelihood ratio tests, lme4 for mixed-effects models, and msm for standard error calculations. Notably, a formal assessment of publication bias was not included in this meta-analysis. This decision was made after considering the complexities and potential low statistical power associated with assessing publication bias in diagnostic accuracy reviews [15, 16].

### Configurations of the generative pre-trained transformer

This research employed specialized configurations of the GPT-4, meticulously engineered through advanced prompt structuring and schema modifications to address distinct investigatory requisites. The spectrum of tasks encompassed the precision refinement of R scripts dedicated to statistical examination, validation of data integrity extracted from included studies against their primary sources, designing comprehensive literature surveys, and enhancement of manuscript vernacular.

## Results

### Study selection process

**Fig 1:**
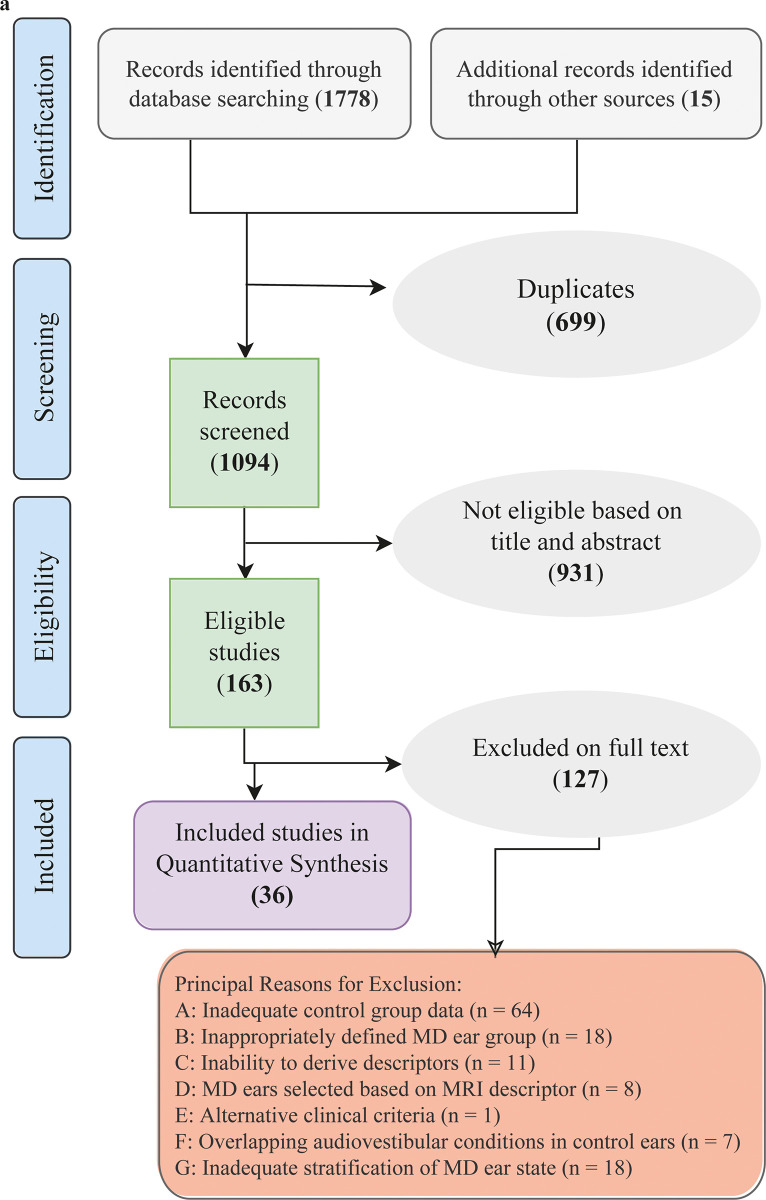
PRISMA flowchart of study selection process. This flowchart illustrates the systematic process of study selection, adhering to the PRISMA guidelines.

Following PRISMA guidelines, our systematic search across databases yielded 1,793 records, with 699 duplicates removed during screening (Fig 1). A total of 1,094 records were scrutinized for relevance, leading to 931 exclusions. The remaining 163 articles underwent full-text review, resulting in 127 further exclusions due to non-conformity with our inclusion criteria, leaving 35 studies for the final quantitative synthesis (Table 1). In this study, no missing data were encountered. This outcome is attributed to the strict inclusion and exclusion criteria applied during the study selection process, which ensured that only studies with complete and relevant data were included in the final analysis.

**Table 1:** An overview of hydrops imaging in MD across diverse MRI grading systems.

Author	MD criteria	Gd contrast	Hydrops Grading	Non-EH ears	Grade1 EH ears	Grade2 EH ears
**Bernaerts 2019 [19]**	Barany	IV	PLE	dMD: 27, Asymp: 76	dMD: 51, Asymp: 2	NC
**Bernaerts 2022 [20]**	Barany	IV	Baráth	dMD: 3, Asymp: 27	dMD: 26, Asymp: 2	dMD: 17, Asymp: 0
**Chen 2021 [21]**	AAO-HNS	IV	Nakashima	dMD: 31, Asymp: 69	dMD: 39, Asymp: 1	dMD: 20, Asymp: 0
**Connor 2022 [22]**	Barany	IV	Nakashima	dMD: 2, Asymp: 19	dMD: 33, Asymp: 16	dMD: 30, Asymp: 2
**Conte 2018 [23]**	Barany	IV	Nakashima	dMD: 7, Asymp: 18, Normal: 23	dMD: 20, Asymp: 5, Normal: 1	NC
**Domínguez 2021 [24]**	Barany	IV	Nakashima	dMD: 15, Asymp: 72	dMD: 68, Asymp: 11	dMD: 42, Asymp: 1
**Guajardo-Vergara 2022 [25]**	Barany	IV	Nakashima	dMD: 14, Asymp: 66	dMD: 62, Asymp: 10	dMD: 37, Asymp: 1
**Han 2022 [26]**	AAO-HNS	IV	Nakashima	dMD: 34, Asymp: 113, pMD: 43	dMD: 46, Asymp: 4, pMD: 6	dMD: 13, Asymp: 0, pMD: 1
**Jasińska 2022 [27]**	Barany	IV	Baráth	dMD: 7, Asymp: 38	dMD: 31, Asymp: 0	dMD: 6, Asymp: 0
**Kahn 2019a [28]**	Barany	IV	Kahn	dMD: 4, Asymp: 24, Normal: 52	dMD: 31, Asymp: 3, Normal: 0	NC
**Kahn 2019b [28]**	Barany	IV	PLE	dMD: 32, Asymp: 27, Normal: 52	dMD: 3, Asymp: 0, Normal: 0	NC
**Kazemi 2022 [29]**	Barany	IV	Baráth	dMD: 5, Asymp: 13, Normal: 22	dMD: 19, Asymp: 1, Normal: 7	dMD: 8, Asymp: 0, Normal: 0
**Kenis 2021a [30]**	Barany	IV	Baráth	dMD: 1, Asymp: 8, pMD: 2	dMD: 4, Asymp: 1, pMD: 2	dMD: 3, Asymp: 1, pMD: 1
**Kenis 2021b [30]**	Barany	IV	PLE	dMD: 5, Asymp: 9, pMD: 3	dMD: 0, Asymp: 0, pMD: 1	NC
**Kirbac 2022 [31]**	Barany	IV	Baráth	dMD: 1, Asymp: 4	dMD: 15, Asymp: 10	dMD: 4, Asymp: 0
**Li 2020 [32]**	AAO-HNS	IT	Nakashima	dMD: 9, Asymp: 29	dMD: 169, Asymp: 149	dMD: NC, Asymp: NC
**Mainnemarre 2020 [33]**	AAO-HNS	IV	Kahn	dMD: 2, Asymp: 32, pMD: 14	dMD: 36, Asymp: 6, pMD: 4	NC
**Morimoto 2017 [34]**	AAO-HNS	IT	Nakashima	dMD: 7, Asymp: 15	dMD: 46, Asymp: 14	dMD: 38, Asymp: 4
**Morimoto 2020 [35]**	Barany	IV	Nakashima	dMD: 2, Normal: 27	dMD: 24, Normal: 15	dMD: 20, Normal: 5
**Morita 2020 [36]**	Barany	IV	Nakashima	dMD: 5, Asymp: 10	dMD: 25, Asymp: 20	dMD: 13, Asymp: 4
**Naganawa 2014 [37]**	AAO-HNS	IT	Nakashima	dMD: 3, Asymp: 4	dMD: 6, Asymp: 6	dMD: 3, Asymp: 3
**Nahmani 2020 [38]**	AAO-HNS	IV	PLE	dMD: 11, Asymp: 13	dMD: 8, Asymp: 0	NC
**Oh 2021 [39]**	Barany	IV	Nakashima	dMD: 6, pMD: 2	dMD: 9, pMD: 10	dMD: NC, pMD: NC
**Okazaki 2017 [40]**	AAO-HNS	IV	Nakashima	dMD: 0, Asymp: 21, pMD: 1	dMD: 28, Asymp: 16, pMD: 8	dMD: 26, Asymp: 3, pMD: 7
**Pai 2020 [41]**	Barany	IV	Nakashima	dMD: 1, Asymp: 6, pMD: 0	dMD: 23, Asymp: 15, pMD: 6	dMD: 21, Asymp: 7, pMD: 4
**Pyykkö 2013 [5]**	AAO-HNS	IT	Nakashima	dMD: 4, pMD: 62	dMD: 64, pMD: 75	dMD: NC, pMD: NC
**Sano 2012 [42]**	AAO-HNS	IV	Nakashima	dMD: 0, pMD: 0	dMD: 1, pMD: 7	dMD: 1, pMD: 7
**Shi 2018 [43]**	Barany	IV	PLE	dMD: 24, Asymp: 139	dMD: 115, Asymp: 0	NC
**Shiraishi 2020 [44]**	Barany	IV	Kahn	dMD: 8, Asymp: 17	dMD: 12, Asymp: 3	NC
**Sousa 2022 [45]**	Barany	IV	Baráth	dMD: 1, pMD: 6	dMD: 8, pMD: 8	dMD: 4, pMD: 4
**Suárez Vega 2020 [46]**	Barany	IV	Nakashima	dMD: 6, pMD: 18	dMD: 18, pMD: 8	dMD: 10, pMD: 4
**Tagaya 2011 [47]**	AAO-HNS	IV	Nakashima	dMD: 5, Asymp: 6	dMD: 9, Asymp: 2	dMD: 8, Asymp: 0
**van Steekelenburg 2020a [3]**	Barany	IV	Baráth	dMD: 23, Asymp: 146, pMD: 6	dMD: 126, Asymp: 3, pMD: 8	NC
**van Steekelenburg 2020b [3]**	Barany	IV	PLE	dMD: 26, Asymp: 144, pMD: 8	dMD: 123, Asymp: 5, pMD: 6	NC
**Wu 2016 [48]**	AAO-HNS	IT	Nakashima	dMD: 3, Asymp: 45	dMD: 51, Asymp: 9	dMD: NC, Asymp: NC
**Xie 2021 [49]**	Barany	IV	Nakashima	dMD: 5, pMD: 17	dMD: 26, pMD: 3	dMD: 16, pMD: 1
**Yamamoto 2010 [50]**	AAO-HNS	IT	Nakashima	dMD: 1, pMD: 3	dMD: 7, pMD: 7	dMD: 5, pMD: 6
**Yoshida 2018 [51]**	AAO-HNS	IV	Nakashima	dMD: 7, Asymp: 17, Normal: 26	dMD: 45, Asymp: 15, Normal: 16	dMD: 37, Asymp: 5, Normal: 4

The table summarizes the criteria used for diagnosing MD, the type of gadolinium (Gd) contrast administered, and the distribution of non-endolymphatic hydrops (Non-EH) ears, Grade 1 EH ears, and Grade 2 EH ears as classified by different grading systems

### Risk of bias and applicability concerns

In the QUADAS-2 evaluation of the included studies (S4 File), patient selection consistently presented a high risk of bias due to the reliance on case-control designs, which inherently limit population diversity and introduce spectrum bias. The absence of a definitive gold standard for MD diagnosis further compounded this issue, resulting in a high-risk assignment for the reference standard across all studies. However, studies that employed comprehensive clinical diagnostic criteria were assigned a low risk of bias, as this approach ensures a representative patient population. The conduct and interpretation of diagnostic tests were also marked by high risk due to the challenges in blinding within MD cohorts, which heightened the potential for observer bias. Conversely, the use of predefined grading systems in all included studies supported a low-risk assessment in this domain, as standardization minimizes subjective interpretation. Applicability concerns were deemed low, given the consistent use of clinically validated assessment protocols, ensuring that the findings are relevant and generalizable to clinical practice.

### Evaluative synopsis of MRI-based cochlear hydrops grading systems

**Fig 2:**
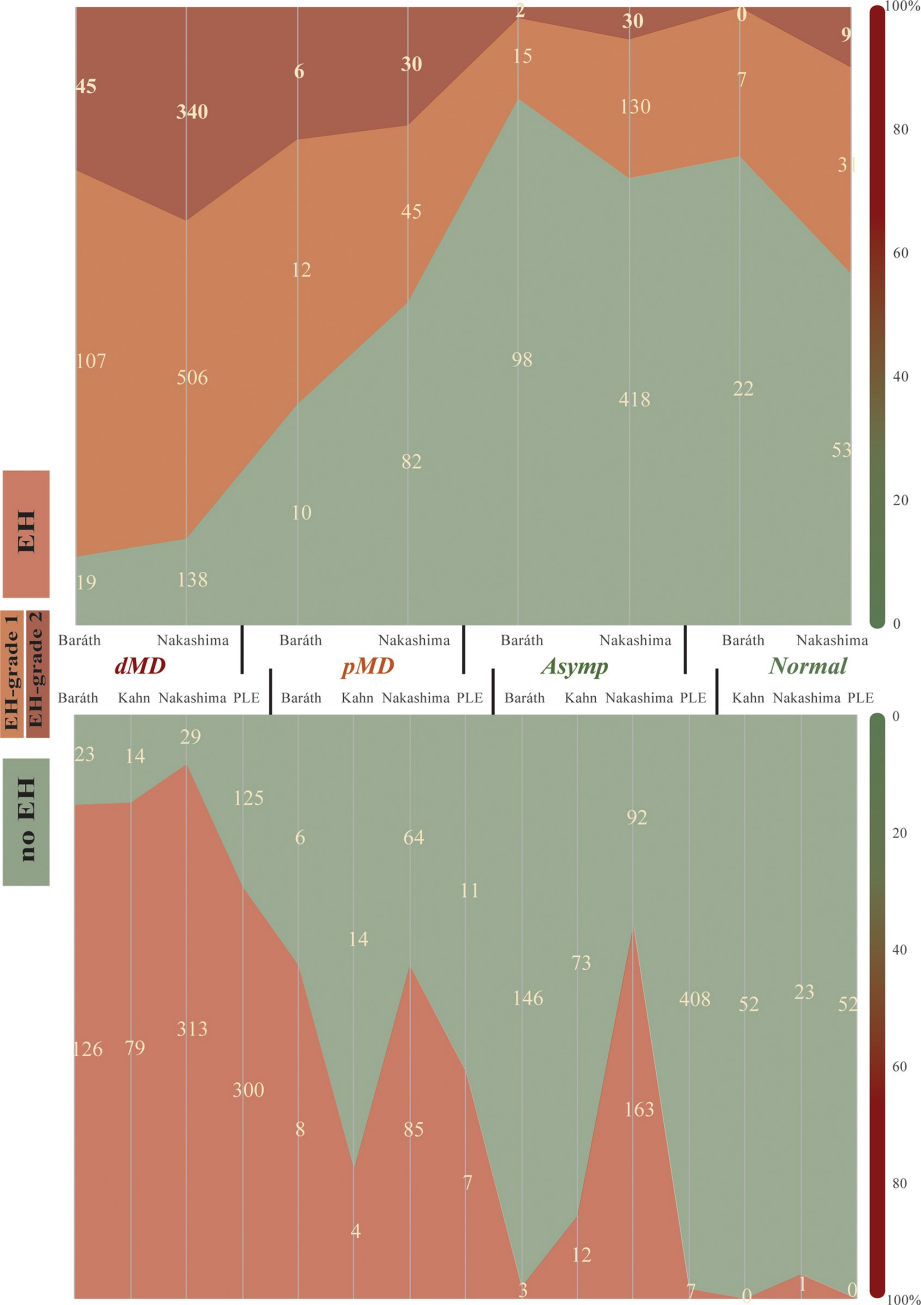
MRI-based cochlear hydrops grading and PLE in MD.

In an analytical review of MRI-based cochlear hydrops classification, the gradated Nakashima and Baráth systems were contrasted with the binary classifications of PLE and Kahn (Table 2). The Nakashima system, focusing on Reissner’s membrane displacement and the scala vestibuli’s spatial dynamics, alongside Baráth’s approach to perilymphatic space dilation, reveals graded hydrops severity in MD staging. Studies utilizing the Baráth system often report fewer cases of Grade 2 hydrops compared to the Nakashima system, which may reflect the stricter criteria for severe hydrops classification in the former. Binary systems of PLE and Kahn focus on the presence of hydrops without grading severity. The aggregated data visualized in Fig 2 further solidifies these interpretations, with the clustered representation of hydrops severity offering a visual corroboration of the textual data. The distribution of hydrops across the ear categories shows a pronounced skew towards severe hydrops in dMD ears.

**Table 2:** Cochlear hydrops classification in MRI systems.

Cochlear classification system	Normal	Grade I	Grade II	INDISTINCT GRADES I AND II
**Nakashima**	No displacement of reissner’s membrane	Displacement of reissner’s membrane, but area of cochlear duct < area of the scala vestibuli	Area of the cochlear duct exceeds the area of the scala vestibuli	-
**Baráth**	None	Mild dilation of the non-enhancing cochlear duct, sparing parts of the enhancing perilymph of the scala vestibuli	The scala vestibuli was uniformly obstructed by the maximally distended cochlear duct	-
**Kahn**	None	The area of the endolymphatic space exceeded the area of the scala vestibuli	-	-
**PLE**	None	Marked perilymphatic enhancement in the basal turn of the cochlea relative to the contralateral	-	-
**Total**	1972	853	462	1108
**PLE**	596	314		
**Kahn**	153	95		
**Nakashima**	799	712	409	562
**Baráth**	324	141	53	137
**Asymp**	1235	145	32	185
**dMD**	356	613	385	818
**Normal**	202	38	9	1
**pMD**	187	57	36	104

This table compares cochlear hydrops gradations—Normal, Grade I (mild), and Grade II (severe)—across MRI systems by Nakashima and Baráth, and single-category systems by PLE and Kahn. It details anatomical descriptions and distributes these gradations among patient groups: asymptomatic (Asymp), definite Meniere’s Disease (dMD), healthy controls (Normal), and probable/possible Meniere’s Disease (pMD).

This figure displays a histogram comparing cochlear hydrops severity grading across MRI systems. The upper portion shows Nakashima and Baráth systems’ grading (Grade I for mild, Grade II for severe hydrops) alongside patient categorizations. The lower part contrasts binary classifications (normal vs. hydrops presence) by PLE and Kahn systems, including data for non-graded studies, across patient categories (asymptomatic, healthy controls, definite, and probable MD).

### Quantitative synthesis of MRI-based EH grading in MD diagnosis

The analysis evaluates the diagnostic performance of MRI-based EH grading, using AAO-HNS and Barany criteria as reference standards (Table 3). At the EH grade 1 cutoff (S1 Fig in S1 File), the Establishment model, which distinguishes dMD from control groups (including normal and asymptomatic subgroups), achieved a sensitivity of 85.4% (CI: 78.5–90.3) and specificity of 82.7% (CI: 78.8–86.0), with a DOR of 27.888 (CI: 16.454–47.268) and a correlation coefficient of -0.076. At the EH grade 2 cutoff (S2 Fig in S1 File), sensitivity increased to 92.1% (CI: 85.9–95.7) with reduced specificity of 70.6% (CI: 64.5–76.1) and a DOR of 28.056 (CI: 14.917–52.770). The Confirmation model (distinguishing dMD from pMD, S3 Fig in S1 File) showed lower diagnostic accuracy, with a DOR of 5.216. For the Spotting model (distinguishing pMD from control, S4 Fig in S1 File), the sensitivity was 48.3% (CI: 34.8–62.1) and specificity 88.0% (CI: 77.8–93.9), with a DOR of 6.882 (CI: 2.725–17.382).

**Table 3:** MRI grading systems’ diagnostic accuracy for MD.

Model estimates	Establishment-dMD vs control (EH grade 1)	Establishment-dMD vs control (EH grade 2)	Confirmation-dMD vs pMD (EH grade 1)	Spotting-pMD vs control (EH grade 1)
**Sensitivity (%95ci)**	0.854 (0.785–0.903)	0.921 (0.859–0.957)	0.744 (0.598–0.851)	0.483 (0.348–0.621)
**Specificity (%95ci)**	0.827 (0.788–0.86)	0.706 (0.645–0.761)	0.642 (0.439–0.805)	0.88 (0.778–0.939)
**Dor (%95ci)**	27.888 (16.454–47.268)	28.056 (14.917–52.77)	5.216 (2.323–11.713)	6.882 (2.725–17.382)
**Var(logit(sen))**	1.512	0.794	1.276	0.143
**Var(logit(spe))**	0.312	0.274	1.567	0.805
**Se(logit(sen))**	0.240	0.330	0.343	0.287
**Se(logit(spec))**	0.128	0.144	0.424	0.380
**Correlation**	-0.076	-0.599	-0.639	-0.184
**Bivariate i2**	0.505	0.387	0.465	0.274
**Area 95% prediction ellipse**	0.272	0.156	0.624	0.366

This table evaluates MRI grading systems’ diagnostic accuracy for MD, presenting sensitivity, specificity, and their 95% confidence intervals (CI), along with Diagnostic Odds Ratio (DOR), variability (Var), standard error (SE) of estimates, and metrics quantifying heterogeneity (Bivariate I^2^) and predictive uncertainty (Area of the 95% Prediction Ellipse).

### Heterogeneity analysis

Heterogeneity was moderate to substantial, indicated by Bivariate I2 values. Predictive uncertainties, shown by Prediction Ellipse areas (Table 3), were smallest in the Establishment model (0.272 for grade 1, 0.156 for grade 2), larger in the Confirmation model (0.624), and moderate in the Spotting model (0.366).

### Subgroup perspective

#### Establishment model

**Fig 3:**
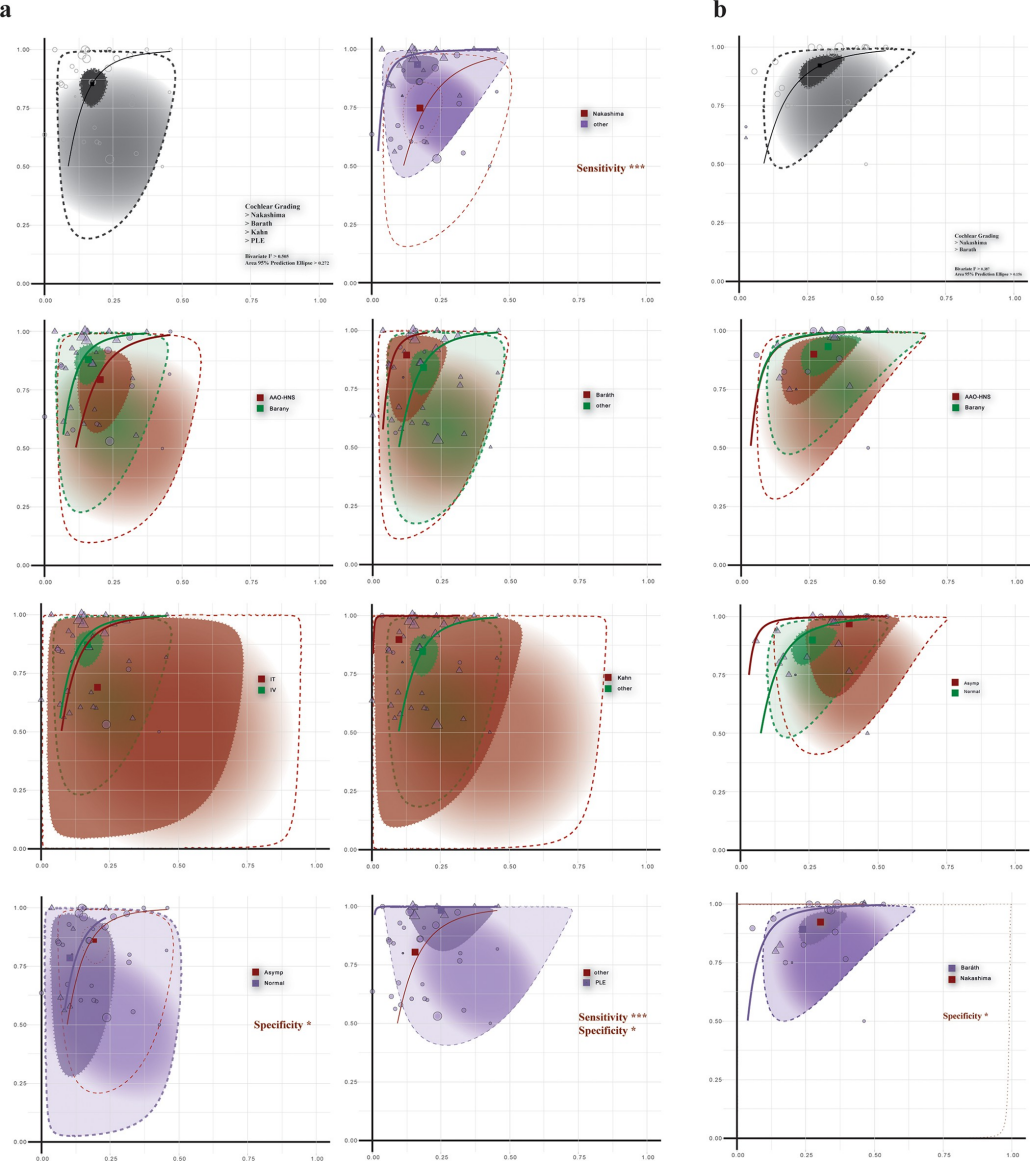
HSROC analysis for MD diagnosis at grade 1 and 2 thresholds. Part (a) examines diagnostic efficacy at Grade 1, showing sensitivity and specificity for various criteria and gadolinium routes. Part (b) compares diagnostic accuracy at Grade 2, assessing criteria performance at this elevated severity. The opening plot of each section shows the aggregate model performance including all subgroups.

As illustrated in the Fig 3 and Table 4, utilizing AAO-HNS criteria as a reference, cochlear EH indicated a sensitivity of 79.6% (CI: 64.9–89.1) and specificity of 79.7% (CI: 72.3–85.6), with a DOR of 15.318 (CI: 6.798–34.515). Barany Society criteria exhibited a higher sensitivity of 88.1% (CI: 80.6–92.9) and specificity of 84.1% (CI: 79.7–87.7), with a DOR of 39.149 (CI: 20.886–73.382), though statistical analysis indicated no significant differences (p-value: sensitivity = 0.182, specificity = 0.253, DOR = 0.188). The subgroup analysis based on gadolinium administration route revealed contrasting outcomes: IT route showed a sensitivity of 68.9% (CI: 39.7–88.2), specificity of 79.4% (CI: 64.5–89.1), and a DOR of 8.556 (CI: 2.205–33.199), while the IV route demonstrated higher sensitivity of 87.1% (CI: 80.5–91.7), specificity of 83.2% (CI: 79.1–86.6), and a DOR of 33.442 (CI: 19.38–57.705), with no significant statistical differences (p-value: sensitivity = 0.098, specificity = 0.545, DOR = 0.196). In the normal ears subgroup (n = 6), a notable specificity of 89.7% (CI: 82.7–94.1) was observed, significantly higher than in the asymptomatic subgroup (p-value = 0.025). Using Nakashima’s criteria (n = 18), a lower sensitivity of 74.9% (CI: 64.3–83.2) was recorded, significantly different from other systems (p-value < 0.001). Barath and Kahn systems showed high sensitivities of 89.5% (CI: 75.1–96.0) and 89.8% (CI: 68.3–97.3), respectively, without significant deviations. The PLE grading system (n = 7) revealed a high sensitivity of 98.4% (CI: 93.7–99.6) but a lower specificity of 74.9% (CI: 65.6–82.3), leading to a remarkably high DOR of 180.207 (CI: 41.13–789.566), significantly outperforming other systems in sensitivity and DOR (p-value < 0.001).

**Table 4:** Diagnostic performance of MRI grading systems in MD subgroups.

Subgroups	Sensitivity (%95CI)	Specificity (%95CI)	DOR (%95CI)
**Establishment model**			
**AAO-HNS (n = 12)**	0.796 (0.649–0.891)	0.797 (0.723–0.856)	15.318 (6.798–34.515)
**Barany (n = 24)**	0.881 (0.806–0.929)	0.841 (0.797–0.877)	39.149 (20.886–73.382)
**P-value**	0.182	0.253	0.188
**IT (n = 4)**	0.689 (0.397–0.882)	0.794 (0.645–0.891)	8.556 (2.205–33.199)
**IV (n = 32)**	0.871 (0.805–0.917)	0.832 (0.791–0.866)	33.442 (19.38–57.705)
**P-value**	0.098	0.545	0.196
**Asymp (n = 30)**	0.86 (0.79–0.91)	0.807 (0.764–0.843)	25.668 (14.48–45.499)
**Normal (n = 6)**	0.785 (0.529–0.922)	0.897 (0.827–0.941)	32.001 (8.353–122.599)
**P-value**	0.430	0.025	0.064
**Nakashima (n = 18)**	0.749 (0.643–0.832)	0.825 (0.768–0.871)	14.119 (7.985–24.966)
**P-value (vs others)**	<0.001	0.815	0.002
**Barath (n = 7)**	0.895 (0.751–0.96)	0.877 (0.799–0.928)	60.646 (19.174–191.815)
**P-value (vs others)**	0.435	0.138	0.226
**Kahn (n = 4)**	0.898 (0.683–0.973)	0.902 (0.811–0.952)	80.764 (16.807–388.105)
**P-value (vs others)**	0.547	0.072	0.155
**PLE (n = 7)**	0.984 (0.937–0.996)	0.749 (0.656–0.823)	180.207 (41.13–789.566)
**P-value (vs others)**	<0.001	0.024	<0.001
**Establishment model- grade 2**			
**AAO-HNS (n = 8)**	0.9 (0.791–0.955)	0.737 (0.645–0.813)	25.146 (10.897–58.027)
**Barany (n = 12)**	0.933 (0.858–0.97)	0.685 (0.606–0.754)	30.152 (13.401–67.841)
**P-value**	0.438	0.375	0.591
**Asymp (n = 17)**	0.924 (0.861–0.96)	0.695 (0.628–0.755)	27.887 (14.291–54.419)
**Normal (n = 3)**	0.893 (0.678–0.97)	0.761 (0.611–0.866)	26.46 (7.007–99.916)
**P-value**	0.606	0.393	0.675
**Nakashima (n = 14)**	0.967 (0.859–0.993)	0.605 (0.501–0.701)	45.442 (9.256–223.084)
**Baráth (n = 6)**	0.898 (0.831–0.94)	0.739 (0.68–0.791)	24.913 (13.922–44.584)
**P-value**	0.118	0.030	0.051
**Confirmation model**			
**AAO-HNS (n = 5)**	0.696 (0.441–0.869)	0.86 (0.669–0.949)	14.075 (5.803–34.136)
**Barany (n = 8)**	0.767 (0.586–0.884)	0.483 (0.293–0.678)	3.07 (1.478–6.378)
**P-value**	0.604	0.010	0.015
**Nakashima (n = 7)**	0.65 (0.455–0.804)	0.716 (0.437–0.891)	4.681 (1.608–13.628)
**P-value (vs others)**	0.108	0.437	0.274
**Barath (n = 3)**	0.76 (0.467–0.92)	0.595 (0.238–0.874)	4.665 (1.045–20.836)
**P-value (vs others)**	0.878	0.773	0.959
**Spotting model**			
**AAO-HNS (n = 3)**	0.444 (0.257–0.647)	0.794 (0.604–0.907)	3.072 (1.293–7.3)
**Barany (n = 5)**	0.582 (0.368–0.769)	0.93 (0.856–0.968)	18.539 (6.78–50.689)
**P-value**	0.320	0.116	0.114
**Nakashima (n = 3)**	0.364 (0.248–0.497)	0.886 (0.884–0.887)	4.423 (2.553–7.663)
**P-value (vs others)**	0.044	0.918	0.131

Presenting a subgroup analysis, this table evaluates MRI systems’ diagnostic performance (Sensitivity, Specificity, DOR, and 95% CIs) in differentiating MD stages. It includes p-values for system comparisons, denoting "vs Others" for one system against the collective. It categorizes patient populations as Asymp, with specific mentions of AAO-HNS criteria and administration routes (IT, IV).

#### Establishment model at grade 2 cutoff

Utilizing the grade 2 cutoff (Fig 3B), AAO-HNS and Barany systems showed comparable results. AAO-HNS criteria demonstrated a sensitivity of 90% (CI: 79.1–95.5) and specificity of 73.7% (CI: 64.5–81.3), closely aligned with the Barany system’s sensitivity of 93.3% (CI: 85.8–97.0) and specificity of 68.5% (CI: 60.6–75.4), yielding DORs of 25.146 (CI: 10.897–58.027) and 30.152 (CI: 13.401–67.841) respectively. The differences were minor and not statistically significant, suggesting their interchangeable utility in clinical assessments at this elevated diagnostic threshold. Nakashima’s criteria, however, exhibited a high sensitivity of 96.7% (CI: 85.9–99.3), albeit at a lower specificity of 60.5% (CI: 50.1–70.1), resulting in a DOR of 45.442 (CI: 9.256–223.084). This contrasts with the Barath system’s sensitivity of 89.8% (CI: 83.1–94.0), and specificity of 73.9% (CI: 68.0–79.1), with a DOR of 24.913 (CI: 13.922–44.584). Notably, statistical analysis indicated a significant difference in specificity (p-value = 0.030) between Nakashima’s and other criteria, marking it as less specific but potentially more sensitive for higher-grade cases.

#### Confirmation model

**Fig 4:**
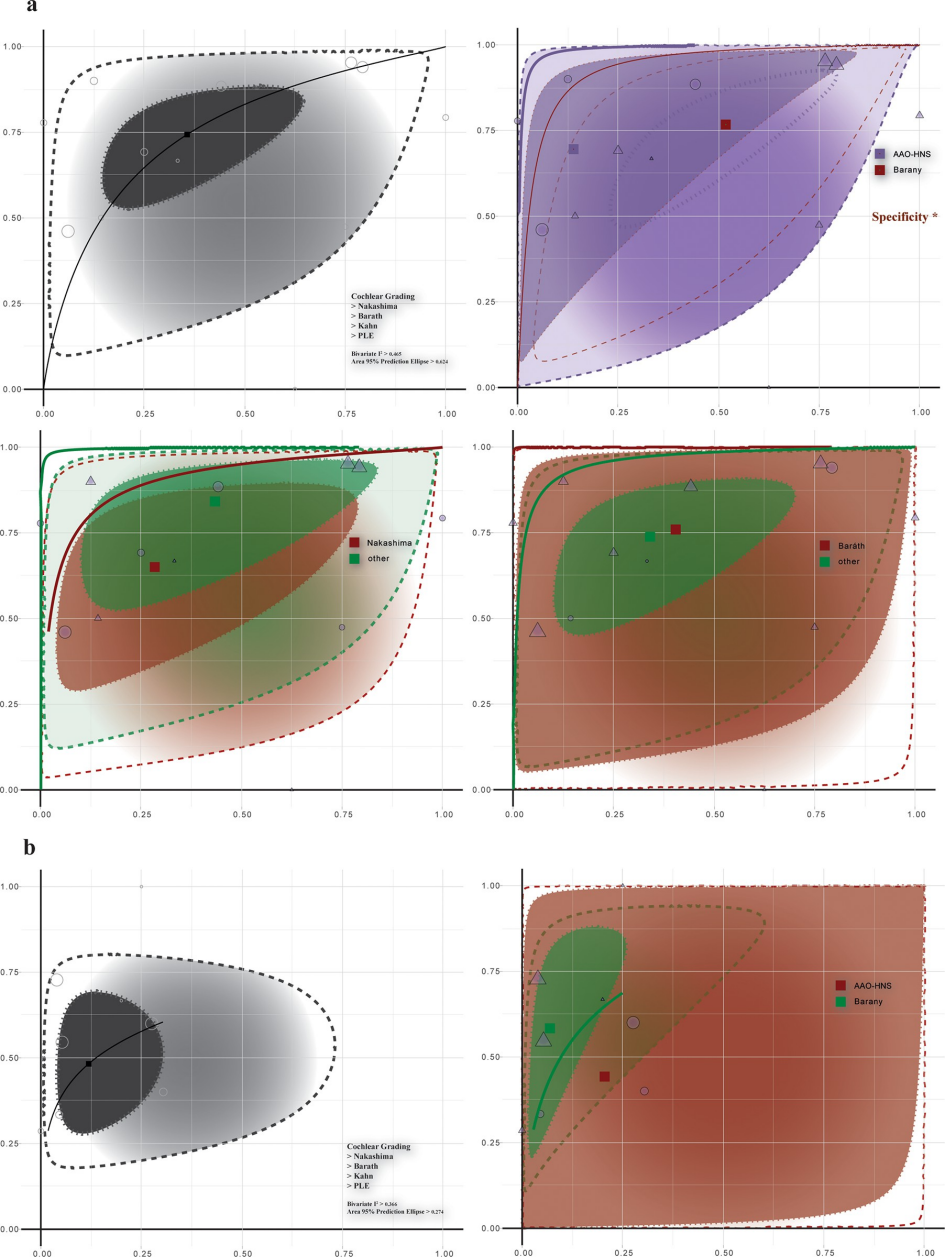
HSROC analysis for MD at grade 1 threshold. Section (a) contrasts definite Meniere’s Disease (dMD) against probable Meniere’s Disease (pMD), while section (b) differentiates probable MD (pMD) from control groups. The opening plot of each section shows the aggregate model performance including all subgroups.

As depicted in Fig 4A, the comparative diagnostic evaluation of dMD versus pMD using AAO-HNS criteria revealed a sensitivity of 69.6% (CI: 44.1–86.9) and a higher specificity of 86.0% (CI: 66.9–94.9), resulting in a DOR of 14.075 (CI: 5.803–34.136). Conversely, the Barany criteria exhibited a sensitivity of 76.7% (CI: 58.6–88.4) but a notably lower specificity of 48.3% (CI: 29.3–67.8), leading to a DOR of 3.07 (CI: 1.478–6.378). The significant disparity in specificity (p-value = 0.010) between these criteria suggests less discriminative capability for distinguishing between dMD and pMD. In the individual analysis of Nakashima versus Barath systems, neither showed significant deviation in sensitivity or DOR from other subgroups. Nakashima’s criteria indicated a sensitivity of 65.0% (CI: 45.5–80.4) and specificity of 71.6% (CI: 43.7–89.1), with a DOR of 4.681 (CI: 1.608–13.628); Barath’s criteria demonstrated a sensitivity of 76.0% (CI: 46.7–92.0) and specificity of 59.5% (CI: 23.8–87.4), with a DOR of 4.665 (CI: 1.045–20.836).

#### Spotting model

In the context of pMD versus control (Fig 4B), using Barany criteria as the reference standard, the cochlear EH grading systems achieved a sensitivity of 58.2% (CI: 36.8–76.9) and specificity of 93.0% (CI: 85.6–96.8), with a DOR of 18.539 (CI: 6.78–50.689). However, statistical significance was not observed. A notable decrease in sensitivity (p-value = 0.044) was seen in the Nakashima system at 36.4% (CI: 24.8–49.7), indicating a reduced efficacy in identifying pMD cases compared to other systems (Table 4).

## Discussion

To be able to monitor the progress of the disease, it is essential to distinguish between the ears at different stages of the disease. Yet, the lack of consensus on grading scales illustrates the broader issues in medical diagnostics, emphasizing the challenges in achieving standardization. Methodologically, the selection of studies for MRI-based EH and perilymphatic space grading systems requires rigorous criteria to synthesize diverse research findings, pointing to the need for methodological refinements to enhance research reliability and validity.

In the quantitative analysis, the Establishment model demonstrated balanced diagnostic potential, whereas the Confirmation and Spotting models showed challenges in differentiating MD stages. In Establishment model, the sensitivity and specificity were high with Grade 1, but specificity reduced with Grade 2, indicating a potential compromise in accurately identifying true negatives at this higher threshold. The Confirmation model grappled with lower sensitivity and specificity in distinguishing dMD from pMD, a reflection of the intrinsic difficulty in separating these closely intertwined MD stages. In the Spotting model, the focus shifts to the detection of pMD, where the overall lower sensitivity, particularly pronounced in the Nakashima system, signals potential risks in overlooking early-stage MD cases, despite the high specificity that underscores the systems’ efficiency in excluding non-MD individuals.

In our meta-analysis, we prioritize an intricate disease probability categorization, rigorous selection criteria, and a discerning adoption of diagnostic standards to address and rectify the heterogeneities and biases pervading prior quantitative syntheses. By striving for a more granular and unified diagnostic schema, our research proposes a clinical framework for applying these grading systems based on disease probability, thus laying the groundwork for improved patient outcomes through more accurate disease staging. Our findings, derived from a rigorous analysis of 35 studies, advocates for a deliberate, informed choice of grading system, aimed at optimizing patient outcomes in the challenging terrain of MD management, reinforcing the indispensable role of customized diagnostic approach, attuned to the clinical objectives.

Grading paradigms confront inconsistencies arising from divergent severity thresholds. The lacuna in standardization not only impedes precise diagnosis and categorization but also exacerbates the interpretative intricacies of MRI outputs, necessitating a more cohesive and multi-faceted approach that reconciles technological proficiency with the complex pathophysiology of MD [17]. The PLE system showed the highest sensitivity and DOR, particularly in the Establishment model. The Nakashima criteria, in contrast, had lower sensitivity, especially notable at the Grade 2 threshold; it also showed a decrease in sensitivity in the Spotting model, highlighting its reduced efficacy in identifying pMD from control cases. The Barath system maintained a balance between sensitivity and specificity across thresholds. The study’s heterogeneity ranged from moderate to substantial, with the smallest predictive uncertainties observed in Establishment models, both at Grade 1 and Grade 2.

The detailed visualization of EH shed light on the intricate pathology of the disease with an unprecedented level of clarity. The detailed approaches, focusing on specific morphological changes such as Reissner’s membrane displacement and perilymphatic space dilation, underscore the complex nature of MD. Conversely, the binary systems, despite their simplicity and ease of use, risk glossing over these subtleties, highlighting the overarching challenge in achieving a diagnostic balance that is both comprehensive and practically applicable.

In the Establishment model, a higher specificity in the normal ears subgroup versus the asymptomatic subgroup accentuates the former’s diagnostic precision in negating MD presence, pivotal for circumventing unwarranted interventions. The PLE system’s paramount sensitivity and DOR, markedly outstripping its counterparts, albeit with a specificity trade-off, positioning it as a potent diagnostic tool in scenarios valuing the maximization of true MD case detection. The exploration of the Establishment model at the Grade 2 cutoff revealed a tightly knit performance between the AAO-HNS and Barany systems, both heralding high sensitivity and moderate specificity, hinting at their interchangeable clinical utility. In the Confirmation model, pronounced specificity disparity between the AAO-HNS and Barany criteria, with the former showcasing a balanced sensitivity and a notably higher specificity, indicated a robust capability to accurately exclude pMD cases. Conversely, the Barany criteria, despite a marginally superior sensitivity, significantly lagged in specificity, unveiling a less discriminative power in segregating dMD from pMD.

The cochlear grading systems varied in their sensitivity and specificity for different comparisons of ear categories. The contrast between quantitative metrics, like Nakashima’s area measurements, and qualitative descriptors, as used by Baráth and PLE, further complicates the task of integrating these systems into a unified diagnostic framework. Additionally, the subjective nature of some systems, particularly those relying on qualitative assessments like Baráth and PLE, introduces the risk of interobserver variability. This can lead to biased or erroneous results, depending on the radiologist’s expertise and interpretive skills. Lastly, a crucial limitation of these grading systems is their failure to account for the dynamic progression of MD and other cochlear pathologies. They offer static snapshots that may not accurately reflect the evolving nature of these conditions.

Several limitations must be acknowledged to fully interpret the findings accurately. Firstly, the diversity in study designs, including participant selection and diagnostic approaches for Meniere’s disease (MD), introduces variability potentially influencing the overall results. Secondly, inherent biases in patient selection, diagnostic criteria, and reporting across studies compromise the integrity of the data. Additionally, the potential for data overrepresentation due to multiple publications by the same authors may introduce bias. Moreover, the lack of standardized diagnostic criteria for MD, the reliance on clinical history for diagnosis, and the limitations of the statistical methods used, such as I^2^ values in diagnostic test accuracy reviews [18], further challenge the interpretation of the findings. These complexities underscore the imperative for rigorous methodological standards, transparent reporting, and advancements towards uniform diagnostic guidelines, thereby enhancing the reliability and validity of mental health research.

## Supporting information

S1 File The forest plots of diagnostic models. (DOCX)

S2 File PRISMA 2009 checklist. (DOCX)

S3 File Reasons for exclusion of database and register reports following full-text review for eligibility. (DOCX)

S4 File Quality Assessment of Diagnostic Accuracy Studies-2 (QUADAS-2). (DOCX)
