# Supplementary material for: The diagnostic performance of cochlear endolymphatic hydrops and perilymphatic enhancement in stratifying Ménière’s disease probabilities: A meta-analysis of semi-quantitative MRI-based grading systems
Source: PLoS One. 2024 Nov 21;19(11):e0310045. doi: 10.1371/journal.pone.0310045 (PMC11581247; doi:10.1371/journal.pone.0310045)

***The diagnostic performance of Cochlear Endolymphatic Hydrops and Perilymphatic Enhancement in Stratifying Ménière's Disease probabilities: A Meta-Analysis of semi-quantitative MRI-based grading systems***

*Supplementary Information*

#
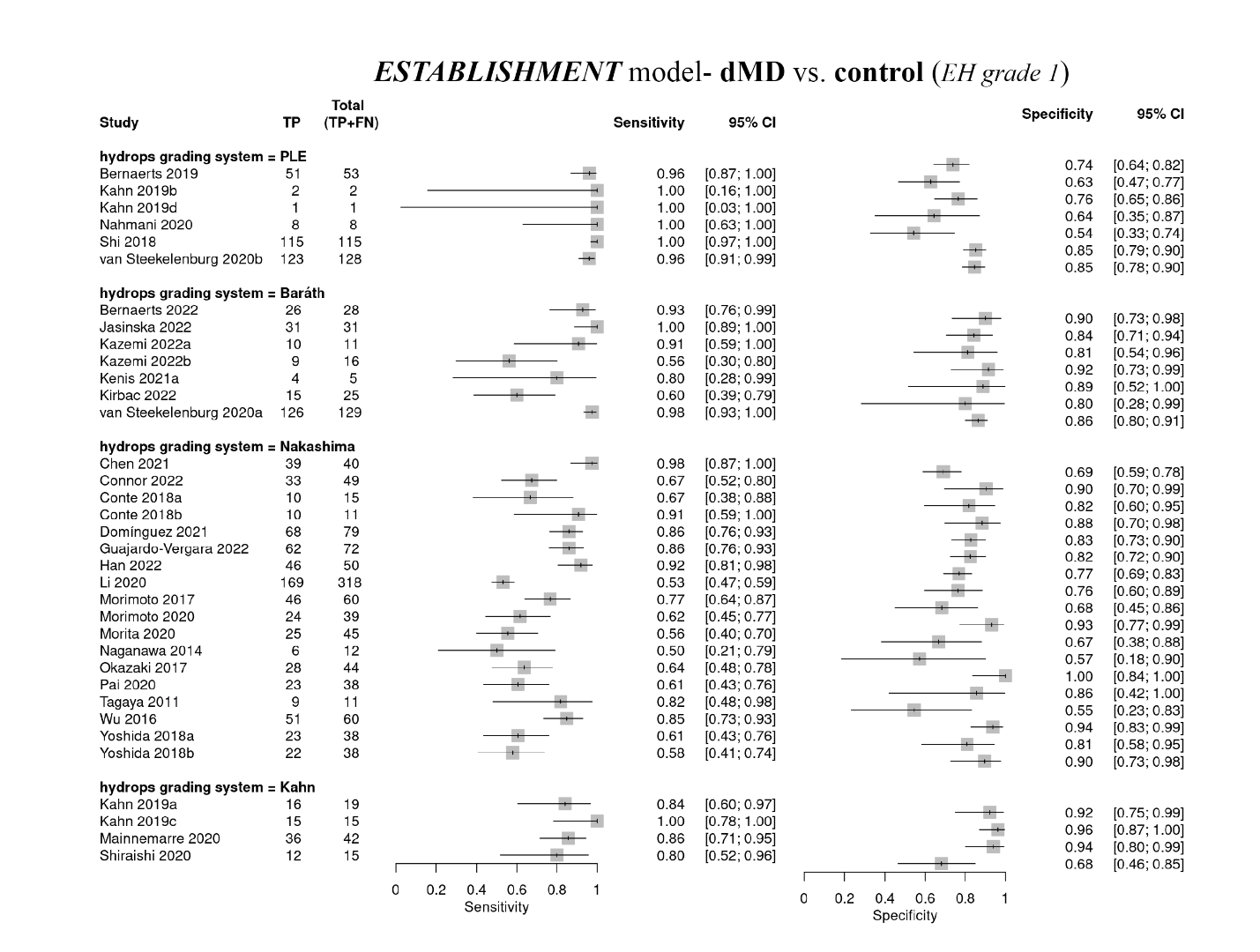
Figure S1: The forest plots of sensitivity and specificity of the dMD vs. control (grade 1) diagnostic model

#
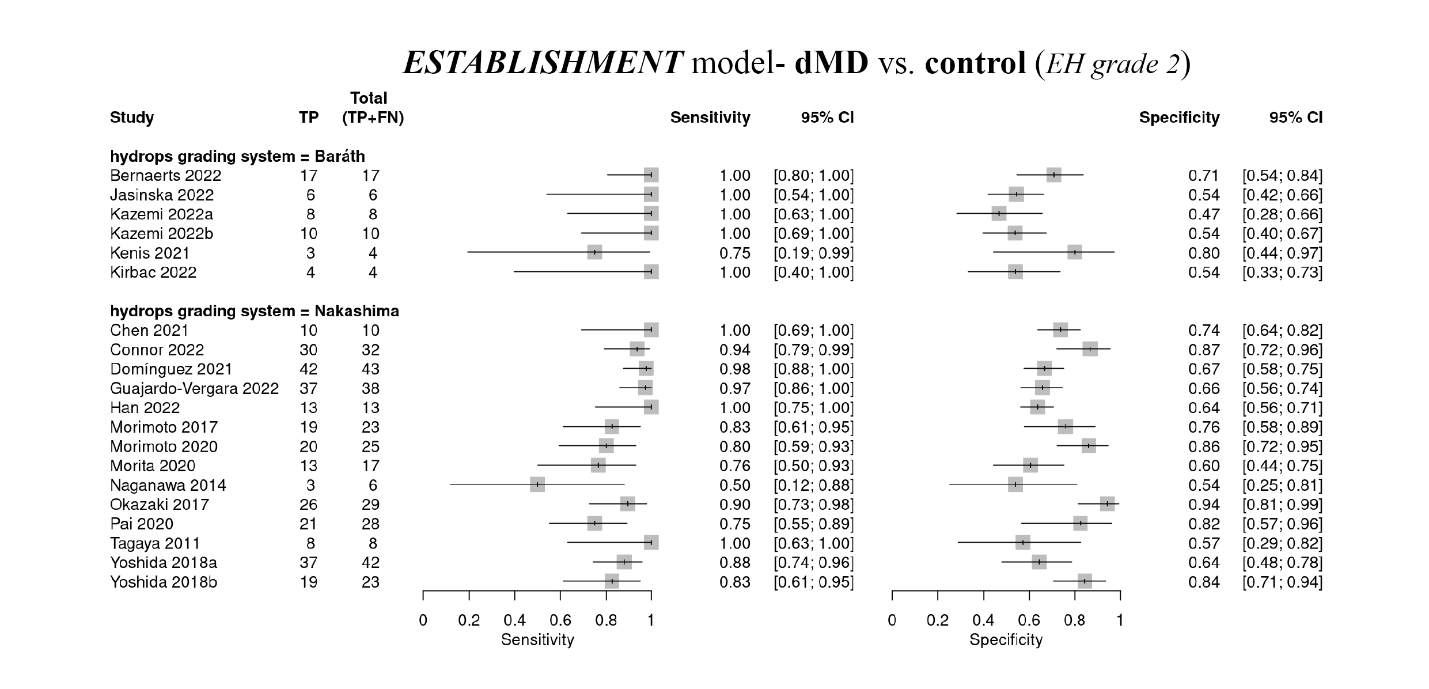
*Figure S2:* The forest plots of sensitivity and specificity of the dMD vs. control (grade 2) diagnostic model

#
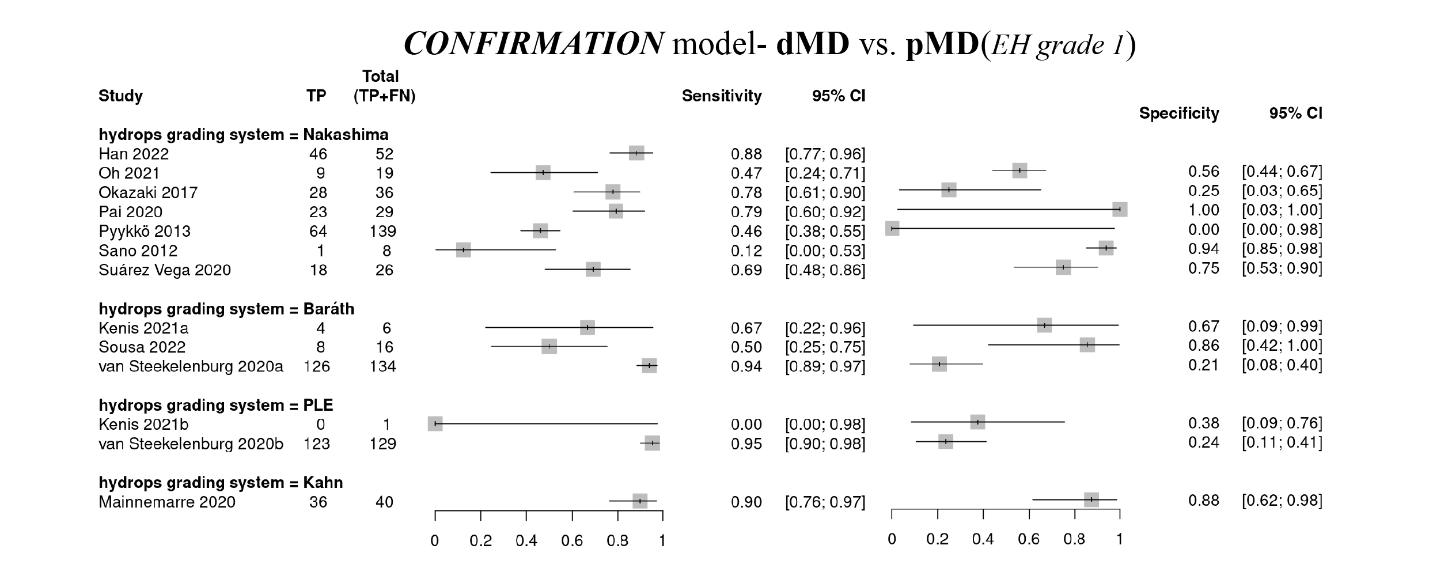
*Figure S3:* The forest plots of sensitivity and specificity of the dMD vs. pMD diagnostic model

# Figure S4: The forest plots of sensitivity and specificity of the pMD vs. control diagnostic model


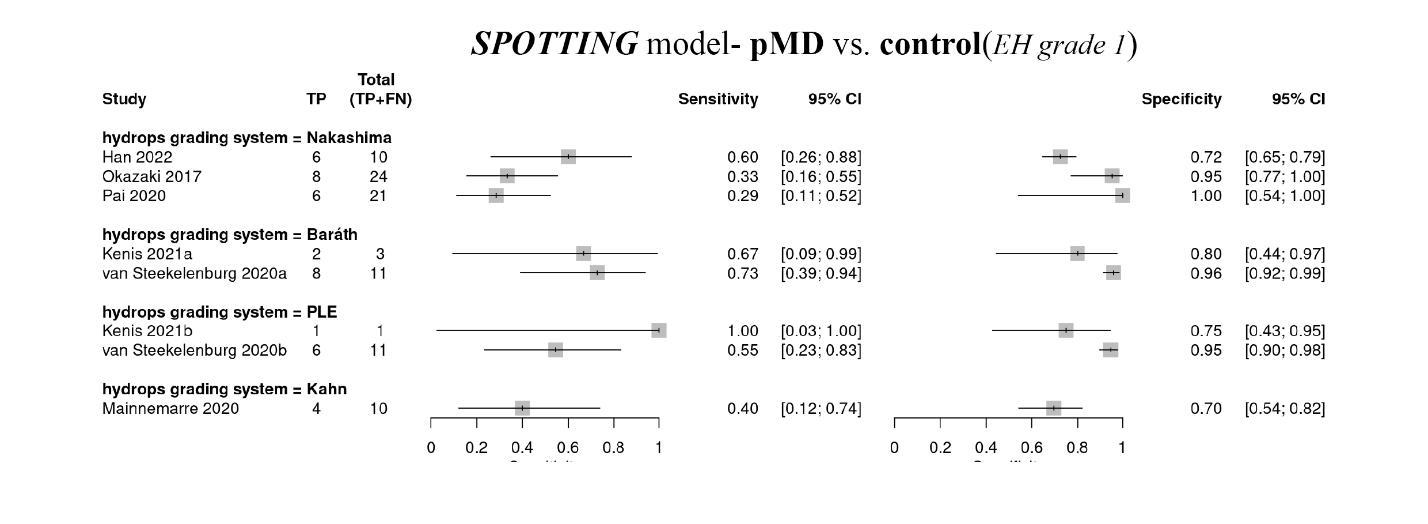

Supplement: S1 File — (DOCX) [file pone.0310045.s001.docx]
