# Supplementary material for: The diagnostic performance of cochlear endolymphatic hydrops and perilymphatic enhancement in stratifying Ménière’s disease probabilities: A meta-analysis of semi-quantitative MRI-based grading systems
Source: PLoS One. 2024 Nov 21;19(11):e0310045. doi: 10.1371/journal.pone.0310045 (PMC11581247; doi:10.1371/journal.pone.0310045)
Supplement: S3 File — (DOCX) [file pone.0310045.s003.docx]

Supporting Information 3: Reasons for Exclusion of Database and Register Reports Following Full-Text Review for Eligibility

Principal Reasons for Exclusion:

A: Absence of data from an appropriate control group.

B: Lack of data from a properly defined Meniere's disease (MD) ear group.

C: Inability to derive semiquantitative/qualitative descriptors from the available quantitative data.

D: MD ears selected based on MRI descriptors rather than clinical criteria.

E: Use of alternative clinical criteria not aligned with Barany Society or AAO-HNS standards.

F: Control ears exhibited overlapping audiovestibular conditions, compromising validity.

G: Inadequate stratification of the MD ear state in the study design.

|  | Alonso JE, Ishiyama GP, Fujiwara RJT et al. Cochlear Meniere’s: A distinct clinical entity with isolated cochlear hydrops on high-Resolution  MRI? Frontiers in Surgery 2021; 8. https://doi.org/10.3389/fsurg.2021.680260 | D |
| --- | --- | --- |
|  | Bachinger D, Eckhard AH, Roosli C, Veraguth D, Huber A, Dalbert A. Endolymphatic hydrops mimicking obstructive Eustachian tube dysfunction: preliminary experience and literature review. European Archives of Oto-Rhino-Laryngology 2021; 278(2), 561–565. https://doi.org/http://dx.doi.org/10.1007/s00405-020-06139-9 | D |
|  | Bier G, Bongers MN, Schabel C, Heindel W, Ernemann U, Hempel JM. In vivo assessment of an endolymphatic hydrops gradient along the cochlea in patients with Menière’s disease by magnetic resonance Imaging — A pilot study. Otol Neurotol 2018; 39(10), e1091–e1099. <https://doi.org/10.1097/MAO.0000000000002016> | A |
|  | Bykowski J, Harris JP, Miller M, Du J , Mafee MF. Intratympanic contrast in the evaluation of Menière disease: Understanding the limits.  American Journal of Neuroradiology 2015; 36(7), 1326–1332. <https://doi.org/10.3174/ajnr.A4277> | A |
|  | Chen W, Geng Y, Luo S, Lin N, Sha Y. The correlation of clinical Features and endolymphatic hydrops visualized by 3D-Real IR MRI in children with sudden sensorineural hearing Loss. Ear, Nose and Throat Journal 2021 <https://doi.org/10.1177/01455613211009432> | B |
|  | Chen W, Wu X, Geng Y, Lin N, Sha Y. The clinical features and image characteristics of Meniere’s disease patients with endolymphatic hydrops confirmed by enhanced magnetic resonance imaging. Brazilian Journal of Otorhinolaryngology 2021 https://doi.org/10.1016/j.bjorl.2021.07.009 | A |
|  | Cho YS, Ahn JM, Choi JE et al. Usefulness of intravenous gadolinium inner ear MR imaging in diagnosis of Ménière’s disease. Scientific  Reports 2018; 8(1). https://doi.org/10.1038/s41598-018-35709-5 | C |
|  | Cho YS, Kim JS, Kim MB et al. Validation of inner ear MRI in patients with Ménière’s disease by comparing endolymphatic hydrops from histopathologic specimens. Scientific Reports 2021; 11(1). <https://doi.org/10.1038/s41598-021-97213-7> | A |
|  | Eliezer M, Toupet M, Housset J, Houdart E, Hautefort C. Recurrent vestibulopathy: are cVEMP, oVEMP and inner ear MRI useful to distinguish patients with Menière's disease and vestibular migraine? Eur Arch Otorhinolaryngol. 2022 Feb;279(2):713-721. doi: 10.1007/s00405-021-06716-6. Epub 2021 Mar 2. PMID: 33651151. | F |
|  | Horii A, Osaki Y, Kitahara T, Imai T, Uno A, Nishiike S, Fujita N, Inohara H. Endolymphatic hydrops in Meniere's disease detected by MRI after intratympanic administration of gadolinium: comparison with sudden deafness. Acta Otolaryngol. 2011 Jun;131(6):602-9. doi: 10.3109/00016489.2010.548403. Epub 2011 Feb 23. PMID: 21344957. | F |
|  | Katayama N, Yamamoto M, Teranishi M, Naganawa S, Nakata S, Sone M, Nakashima T. Relationship between endolymphatic hydrops and vestibular-evoked myogenic potential. Acta Otolaryngol. 2010 Aug;130(8):917-23. doi: 10.3109/00016480903573187. PMID: 20105111. | F |
|  | Kawai H, Naganawa S, Ishihara S, Sone M, Nakashima T. MR imaging of the cochlear modiolus after intratympanic administration of Gd-DTPA. Magn Reson Med Sci. 2010;9(1):23-9. doi: 10.2463/mrms.9.23. PMID: 20339263. | F |
|  | Nakada T, Yoshida T, Suga K, Kato M, Otake H, Kato K, Teranishi M, Sone M, Sugiura S, Kuno K, Pyykkö I, Naganawa S, Watanabe H, Sobue G, Nakashima T. Endolymphatic space size in patients with vestibular migraine and Ménière's disease. J Neurol. 2014 Nov;261(11):2079-84. doi: 10.1007/s00415-014-7458-9. Epub 2014 Aug 7. PMID: 25099513. | F |
|  | Sun W, Guo P, Ren T, Wang W. Magnetic resonance imaging of intratympanic gadolinium helps differentiate vestibular migraine from Ménière disease. Laryngoscope. 2017 Oct;127(10):2382-2388. doi: 10.1002/lary.26518. Epub 2017 Feb 21. PMID: 28220492. | F |
|  | Qin, H., He, B., Wu, H., Li, Y., Chen, J., Wang, W., Zhang, F., Duan, M., & Yang, J. (2021). Visualization of Endolymphatic Hydrops in Patients With Unilateral Idiopathic Sudden Sensorineural Hearing Loss With Four Types According to Chinese Criterion. Frontiers in surgery, 8, 682245. https://doi.org/10.3389/fsurg.2021.682245 | F |
|  | Shimono M, Teranishi M, Yoshida T, Kato M, Sano R, Otake H, Kato K, Sone M, Ohmiya N, Naganawa S, Nakashima T. Endolymphatic hydrops revealed by magnetic resonance imaging in patients with acute low-tone sensorineural hearing loss. Otol Neurotol. 2013 Sep;34(7):1241-6. doi: 10.1097/MAO.0b013e3182990e81. PMID: 23921924. | E |
|  | Baráth K, Schuknecht B, Naldi AM, Schrepfer T, Bockisch CJ, Hegemann SC. Detection and grading of endolymphatic hydrops in Menière disease using MR imaging. AJNR Am J Neuroradiol. 2014 Jul;35(7):1387-92. doi: 10.3174/ajnr.A3856. Epub 2014 Feb 13. PMID: 24524921; PMCID: PMC7966587. | G |
|  | Grieve SM, Obholzer R, Malitz N, Gibson WP, Parker GD. Imaging of endolymphatic hydrops in Meniere's disease at 1.5 T using phase-sensitive inversion recovery: (1) demonstration of feasibility and (2) overcoming the limitations of variable gadolinium absorption. Eur J Radiol. 2012 Feb;81(2):331-8. doi: 10.1016/j.ejrad.2011.01.073. Epub 2011 Feb 16. PMID: 21330087. | G |
|  | Carfrae MJ, Holtzman A, Eames F, Parnes SM, Lupinetti A. 3 Tesla delayed contrast magnetic resonance imaging evaluation of Ménière's disease. Laryngoscope. 2008 Mar;118(3):501-5. doi: 10.1097/MLG.0b013e31815c1a61. PMID: 18091332. | G |
|  | Boegle R, Gerb J, Kierig E et al (2021) Intravenous delayed gado-  linium -enhanced MR imaging of the endolymphatic space: a  methodological comparative study. Front Neurol 12:647296 | G |
|  | Choi JE, Kim YK, Cho YS, Lee K, Park HW, Yoon SH, Kim HJ, Chung WH. Morphological correlation between caloric tests and vestibular hydrops in Ménière's disease using intravenous Gd enhanced inner ear MRI. PLoS One. 2017 Nov 30;12(11):e0188301. doi: 10.1371/journal.pone.0188301. PMID: 29190293; PMCID: PMC5708622. | G |
|  | Eliezer M, Poillon G, Horion J, Lelion P, Gerardin E, Magne N, Gillibert A, Attyé A. MRI diagnosis of saccular hydrops: Comparison of heavily-T2 FIESTA-C and 3D-FLAIR sequences with delayed acquisition. J Neuroradiol. 2021 Nov;48(6):446-452. doi: 10.1016/j.neurad.2019.04.005. Epub 2019 Apr 26. PMID: 31034895. | G |
|  | Eliezer M, Poillon G, Gillibert A, Horion J, Cruypeninck Y, Gerardin E, Magne N, Attyé A. Comparison of enhancement of the vestibular perilymph between gadoterate meglumine and gadobutrol at 3-Tesla in Meniere's disease. Diagn Interv Imaging. 2018 May;99(5):271-277. doi: 10.1016/j.diii.2018.01.002. Epub 2018 Feb 15. PMID: 29398574. | G |
|  | Grosser D, Willenborg K, Dellani P, Avallone E, Götz F, Böthig D, Warnecke A, Lanfermann H, Lenarz T, Giesemann A. Vestibular Aqueduct Size Correlates With the Degree of Cochlear Hydrops in Patients With and Without Menière's Disease. Otol Neurotol. 2021 Dec 1;42(10):e1532-e1536. doi: 10.1097/MAO.0000000000003300. PMID: 34766949. | G |
|  | Ito T, Kitahara T, Inui H, Miyasaka T, Kichikawa K, Ota I, Nario K, Matsumura Y, Yamanaka T. Endolymphatic space size in patients with Meniere's disease and healthy controls. Acta Otolaryngol. 2016 Sep;136(9):879-82. doi: 10.3109/00016489.2016.1169556. Epub 2016 Apr 15. PMID: 27080254. | G |
|  | Imai T, Uno A, Kitahara T, Okumura T, Horii A, Ohta Y, Sato T, Okazaki S, Kamakura T, Ozono Y, Watanabe Y, Hanada Y, Imai R, Ohata K, Inohara H. Evaluation of endolymphatic hydrops using 3-T MRI after intravenous gadolinium injection. Eur Arch Otorhinolaryngol. 2017 Dec;274(12):4103-4111. doi: 10.1007/s00405-017-4739-9. Epub 2017 Sep 25. PMID: 28948373. | G |
|  | Hagiwara M, Roland JT Jr, Wu X, Nusbaum A, Babb JS, Roehm PC, Hammerschlag P, Lalwani AK, Fatterpekar G. Identification of endolymphatic hydrops in Ménière's disease utilizing delayed postcontrast 3D FLAIR and fused 3D FLAIR and CISS color maps. Otol Neurotol. 2014 Dec;35(10):e337-42. doi: 10.1097/MAO.0000000000000585. PMID: 25251300. | G |
|  | Murofushi T, Tsubota M, Kanai Y, Endo H, Ushio M. Association of cervical vestibular-evoked myogenic potential tuning property test results with MRI findings of endolymphatic hydrops in Meniere's disease. Eur Arch Otorhinolaryngol. 2021 Sep;278(9):3267-3273. doi: 10.1007/s00405-020-06410-z. Epub 2020 Oct 9. PMID: 33037440. | G |
|  | Lin KT, Lu CJ, Young YH. Magnetic resonance imaging: Role on diagnosing all types of endolymphatic hydrops. J Formos Med Assoc. 2022 Jul;121(7):1325-1333. doi: 10.1016/j.jfma.2021.08.027. Epub 2021 Oct 20. PMID: 34686411. | G |
|  | Naganawa S, Yamazaki M, Kawai H, Bokura K, Sone M, Nakashima T. Imaging of endolymphatic and perilymphatic fluid after intravenous administration of single-dose gadodiamide. Magn Reson Med Sci. 2012;11(2):145-50. doi: 10.2463/mrms.11.145. PMID: 22790302. | G |
|  | Naganawa S, Yamazaki M, Kawai H, Bokura K, Sone M, Nakashima T. Three-dimensional visualization of endolymphatic hydrops after intravenous administration of single-dose gadodiamide. Magn Reson Med Sci. 2013;12(2):147-51. doi: 10.2463/mrms.2012-0050. Epub 2013 May 10. PMID: 23666157. | G |
|  | Tuñón Gómez M, Lobo Duro DR, Brea Álvarez B, García-Berrocal JR. Diagnosis of endolymphatic hydrops by means of 3T magnetic resonance imaging after intratympanic administration of gadolinium. Radiologia. 2017 Mar-Apr;59(2):159-165. English, Spanish. doi: 10.1016/j.rx.2016.10.006. Epub 2016 Dec 23. PMID: 28017456. | G |
|  | Tanigawa T, Tamaki T, Yamamuro O, Tanaka H, Nonoyama H, Shiga A, Sato T, Ueda H. Visualization of endolymphatic hydrops after administration of a standard dose of an intravenous gadolinium-based contrast agent. Acta Otolaryngol. 2011 Jun;131(6):596-601. doi: 10.3109/00016489.2010.548402. Epub 2011 Feb 25. PMID: 21351819. | G |
|  | Yoshida T, Kobayashi M, Sugimoto S, Teranishi M, Naganawa S, Sone M. Evaluation of the blood-perilymph barrier in ears with endolymphatic hydrops. Acta Otolaryngol. 2021 Aug;141(8):736-741. doi: 10.1080/00016489.2021.1957500. Epub 2021 Aug 4. PMID: 34346271. | G |
|  | Fiorino F, Pizzini FB, Beltramello A, Barbieri F. MRI performed after intratympanic gadolinium administration in patients with Ménière disease: Correlation with symptoms and signs. European Archives of Oto-Rhino-Laryngology 2011; 268(2), 181–187.  <https://doi.org/10.1007/s00405-010-1353-5> | A |
|  | Fiorino F, Pizzini FB, Barbieri F, Beltramello A. Variability in the perilymphatic diffusion of gadolinium does not predict the outcome of intratympanic gentamicin in patients with Ménière’s disease. Laryngoscope 2012; 122(4): 907–911. https://doi.org/10.1002/lary.23211 | A |
|  | Fiorino F, Mattellini B, Vento M, Mazzocchin L, Bianconi L, Pizzini FB. Does the intravenous administration of frusemide reduce endolymphatic hydrops? Journal of Laryngology and Otology 2016; 130(3): 242–247. https://doi.org/10.1017/S0022215115003527 | A |
|  | Fukuoka H, Tsukada K, Miyagawa M et al. Semi-quantitative evaluation of endolymphatic hydrops by bilateral intratympanic gadoliniumbased contrast agent (GBCA) administration with MRI for Meniere’s disease. Acta Oto-Laryngologica 2010; 130(1): 10–16.  https://doi.org/10.3109/00016480902858881 | C |
|  | Fukuoka H, Takumi Y, Tsukada K et al. Comparison of the diagnostic value of 3 T MRI after intratympanic injection of GBCA, electrocochleography, and the glycerol test in patients with Meniere’s disease. Acta Oto-Laryngologica 2010; 132(2): 141–145.  https://doi.org/10.3109/00016489.2011.635383 | C |
|  | Fukushima M, Ito R, Miyaguchi S et al. Preceding profound deafness and co-factors promote development of endolymphatic hydrops in preliminary patients with delayed endolymphatic hydrops. Acta Oto-Laryngologica 2016; 136(12): 1304–1308.  https://doi.org/10.1080/00016489.2016.1203993 | B |
|  | Fukushima M, Oya R, Akazawa H, Tsuruta Y, Inohara H. Gadolinium-enhanced inner ear magnetic resonance imaging for evaluation of delayed endolymphatic hydrops, including a bilateral case. Acta Oto-Laryngologica 2016; 136(5): 451–455.  https://doi.org/10.3109/00016489.2015.1129554 | A |
|  | Fukushima M, Kitahara T, Oya R, Akahani S, Inohara H, Naganawa S ,Takeda N. Longitudinal up-regulation of endolymphatic hydrops in patients with Meniere’s disease during medical treatment. Laryngoscope Investigative Otolaryngology 2017; 2(6): 344–350.  https://doi.org/10.1002/lio2.115 | C |
|  | Fukushima M, Yokoi K, Iga J, Akahani S, Inohara H, Takeda N. Contralateral type of delayed endolymphatic hydrops may consist of two phenotypes based on a magnetic resonance imaging preliminary study. Acta Oto-Laryngologica 2017; 137(11): 1153–1157.  https://doi.org/10.1080/00016489.2017.1347825 | B |
|  | Gu X, Fang ZM, Liu Y, Lin SL, Han B, Zhang R, ChenVX. Diagnostic value of three-dimensional magnetic resonance imaging of inner ear after intratympanic gadolinium injection, and clinical application of magnetic resonance imaging scoring system in patients with delayed endolymphatic hydrops. Journal of Laryngology and Otology 2014; 128(1): 53–59. https://doi.org/10.1017/S0022215113003289 | A |
|  | Gu X, Fang ZM, Liu Y, Huang ZW, Zhang R, Chen X. Diagnostic advantages of intratympanically gadolinium contrast-enhanced magnetic resonance imaging in patients with bilateral Meniere’s disease. American Journal of Otolaryngology - Head and Neck Medicine and  Surgery 2015; 36(1): 67–73. https://doi.org/10.1016/j.amjoto.2014.10.003 | A |
|  | Guo P, Sun W, Shi S, Zhang F, Wang J, Wang W. Quantitative evaluation of endolymphatic hydrops with MRI through intravenous gadolinium administration and VEMP in unilateral definite Meniere’s disease. European Archives of Oto-Rhino-Laryngology 2019; 276(4): 993–1000. https://doi.org/10.1007/s00405-018-05267-7 | A |
|  | Gürkov R, Flatz W, Louza J, Strupp M, Ertl-Wagner B, Krause E. In vivo visualized endolymphatic hydrops and inner ear functions in patients with electrocochleographically confirmed Ménière’s disease. Otol Neurotol 2012; 33: 1040-45. | A |
|  | Gürkov R, Flatz W, Keeser D, Strupp M, Ertl-Wagner B, Krause E. Effect of standard-dose betahistine on endolymphatic hydrops: An MRI pilot study. European Archives of Oto-Rhino-Laryngology 2013;270(4), 1231–1235. https://doi.org/10.1007/s00405-012-2087-3 | A |
|  | Gürkov R , Flatz W, Ertl-Wagner B, Krause E. Endolymphatic hydrops in the horizontal semicircular canal: A morphologic correlate for canal paresis in Ménière’s disease. Laryngoscope 2013; 123(2), 503–506. https://doi.org/10.1002/lary.23395 | A |
|  | Gürkov R, Berman A , Dietrich O et al. MR volumetric assessment of endolymphatic hydrops. European Radiology 2015; 25(2), 585–595.  https://doi.org/10.1007/s00330-014-3414-4 | A |
|  | He J, Peng A, Hu J et al. Dynamics in endolymphatic hydrops and symptoms in Meniere’s disease after endolymphatic duct blockage, preliminary results. Frontiers in Neurology 2021; 11. <https://doi.org/10.3389/fneur.2020.622760> | A |
|  | He B, Zhang F, Zheng H et al. The correlation of a 2D volume-referencing endolymphatic-hydrops grading system with extra-tympanic electrocochleography in patients with definite Ménière’s disease. Frontiers in Neurology 2021; 11.  <https://doi.org/10.3389/fneur.2020.595038> | A |
|  | Heider C, Plontke S, Gotze G, Rahne T, Kosling S. Clinical application of MR imaging with contrast-based 3D IR-sequence at Meniere  Disease. Laryngol-Rhino-Otologie Conference HNO 2018; 97 (suppl 2):S172-S173 | A |
|  | Higashi-Shingai K, Imai T, Okumura T et al. Change in endolymphatic hydrops 2 years after endolymphatic sac surgery evaluated by MRI.  Auris Nasus Larynx 2019; 46(3): 335–345. <https://doi.org/10.1016/j.anl.2018.10.011> | A |
|  | Homann G, Vieth V, Weiss D, Nikolaou K, Heindel W, Notohamiprodjo M, Böckenfeld Y. Semi-quantitative vs. volumetric determination of endolymphatic space in Menière’s disease using endolymphatic hydrops 3T-HR-MRI after intravenous gadolinium injection. PLoS ONE 2015; 10(3). https://doi.org/10.1371/journal.pone.0120357 | B |
|  | Hornibrook J, Coates M, Goh A, Gourley J, Bird P. Magnetic resonance imaging for Ménière’s disease: Correlation with tone burst electrocochleography. Journal of Laryngology and Otology 2012; 126(2): 136–141. https://doi.org/10.1017/S0022215111003112 | A |
|  | Hornibrook J, Flook E, Greig S et al. MRI inner ear imaging and tone burst electrocochleography in the diagnosis of Ménière’s disease. Otol  Neurotol 2015; 36 (6):1109-1114. doi: [10.1097/mao.0000000000000782](https://doi.org/10.1097/mao.0000000000000782) | A |
|  | Inui H, Sakamoto T, Ito T, Kitahara T. Magnetic resonance imaging of endolymphatic space in patients with sensorineural hearing loss: comparison between fluctuating and idiopathic sudden sensorineural hearing loss. Acta Oto-Laryngologica 2020; 140(5): 345–350.  https://doi.org/10.1080/00016489.2020.1720919 | C |
|  | Ito T, Inui H, Miyasaka T et al. Relationship between changes in hearing function and volumes of endolymphatic hydrops after endolymphatic sac drainage. Acta Oto-Laryngologica 2019; 139(9): 739–746. <https://doi.org/10.1080/00016489.2019.1630757> | A |
|  | Ito T, Inui H, Miyasaka T et al. Endolymphatic volume in patients with Meniere’s disease and healthy controls: Three-dimensional analysis with magnetic resonance imaging. Laryngoscope Investigative Otolaryngology 2019; 4(6): 653–658. https://doi.org/10.1002/lio2.313 | C |
|  | Ito T, Inui H, Miyasaka T et al. Three-dimensional magnetic resonance imaging reveals the relationship between the control of vertigo and decreases in endolymphatic hydrops after endolymphatic sac drainage with steroids for Meniere’s disease. Frontiers in Neurology 2019; 10. https://doi.org/10.3389/fneur.2019.00046 | A |
|  | Ito T, Inoue T, Inui H et al. Novel magnetic resonance imaging-based method for accurate diagnosis of Meniere’s disease. Frontiers in  Surgery 2021; 8. https://doi.org/10.3389/fsurg.2021.671624 | C |
|  | Iwasa Y-I, Tsukada K, Kobayashi M et al. Bilateral delayed endolymphatic hydrops evaluated by bilateral intratympanic injection of gadodiamide with 3T-MRI. PLoS ONE 2018; 13(12). https://doi.org/10.1371/journal.pone.0206891 | B |
|  | Kato K, Yoshida T, Teranishi M et al. Peak width in multifrequency tympanometry and endolymphatic hydrops revealed by magnetic resonance imaging. Otol Neurotol 2012; 33(6): 912-5.doi: [10.1097/MAO.0b013e31825d9a72](https://doi.org/10.1097/mao.0b013e31825d9a72) | A |
|  | Kato M, Sugiura M, Shimono M et al. Endolymphatic hydrops revealed by magnetic resonance imaging in patients with atypical Meniere’s disease. Acta Oto-Laryngologica 2013 133(2): 123–129. https://doi.org/10.3109/00016489.2012.726374 | A |
|  | Kato M, Teranishi M, Katayama N, Sone M, Naganawa S, Nakashima T. Association between endolymphatic hydrops as revealed by magnetic resonance imaging and caloric response. Otology Neurotol 32(9): 1480-5. doi:[10.1097/MAO.0b013e318235568d](https://doi.org/10.1097/mao.0b013e318235568d) | A |
|  | Kim TY, Park DW, Lee YJ, Lee JY, Lee SH, Chung JH, Lee S. Comparison of inner ear contrast enhancement among patients with unilateral inner ear symptoms in MR images obtained 10 minutes and 4 hours after gadolinium injection. AJNR 2015; 36(12): 2367–2372.  https://doi.org/10.3174/ajnr.A4439 | B |
|  | Kirsch V, Nejatbakhshesfahani F, Ahmadi SA, Dieterich M, Ertl-Wagner B. A probabilistic atlas of the human inner ear’s bony labyrinth enables reliable atlas-based segmentation of the total fluid space. Journal of Neurology 2019; 266: 52–61.  https://doi.org/10.1007/s00415-019-09488-6 | D |
|  | Kitano K, Kitahara T, Ito T, Shiozaki T, Wada Y, Yamanaka T. Results in caloric test, video head impulse test and inner ear MRI in patients with Ménière’s disease. Auris Nasus Larynx 2020; 47(1): 71–78. https://doi.org/10.1016/j.anl.2019.06.002 | A |
|  | Li Y, Sha Y, Wang F et al. Comprehensive comparison of MR image quality between intratympanic and intravenous gadolinium injection using 3D real IR sequences. Acta Oto-Laryngologica 2019;139(8): 659–664. https://doi.org/10.1080/00016489.2019.1600719 | A |
|  | Liu F, Huang W, Chen Q, Meng X, Wang Z, He Y. Non-invasive evaluation of the effect of endolymphatic sac decompression in Ménière’s disease using magnetic resonance imaging. Acta Oto-Laryngologica 2014; 134(7): 666–671.  https://doi.org/10.3109/00016489.2014.885118 | A |
|  | Louza J, Krause E, Gurkov R. Hearing function after intratympanic application of gadolinium-based contrast agent: A long-term evaluation.  Laryngoscope 2015; 125(10):2366-2370 | A |
|  | Maxwell AK, Ishiyama G, Karnezis S, Ishiyama A. Isolated saccular hydrops on high-resolution MRI is associated with full spectrum  Menière’s Disease. Otology & Neurotology 2021; 42(6): 876–882. https://doi.org/10.1097/MAO.0000000000003051 | D |
|  | Min XH, Gu H, Zhang Y, Li K, Pan ZY, Jiang T. Clinical value of abnormal MRI findings in patients with unilateral sudden sensorineural hearing loss. Diagn Interv Radiol 2020; 26(5): 429-436. doi: 10.5152/dir.2020.19229 | B |
|  | Morioka M, Sugimoto S, Yoshida T et al. Dilatation of the endolymphatic space in the ampulla of the posterior semicircular canal: A new clinical finding detected on magnetic resonance imaging. Otol Neurotol 2021; 42(6) e643–e647.  <https://doi.org/10.1097/MAO.0000000000003073> | D |
|  | Naganawa S, Satake H, Kawamura M, Fukatsu H, Sone M, Nakashima T. Separate visualization of endolymphatic space, perilymphatic space and bone by a single pulse sequence; 3D-inversion recovery imaging utilizing real reconstruction after intratympanic Gd-DTPA administration at 3 Tesla. European Radiology 2008; 18(5): 920–924. https://doi.org/10.1007/s00330-008-0854-8 | A |
|  | Naganawa S, Ishihara S, Iwano S, Sone M, Nakashima T. Three-dimensional (3D) visualization of endolymphatic hydrops after  intratympanic injection of Gd-DTPA: Optimization of a 3D-real inversion-recovery turbo spin-echo (TSE) sequence and application of a 32channel head coil at 3T. Journal of Magnetic Resonance Imaging 2010; 31(1), 210–214. https://doi.org/10.1002/jmri.22012 | B |
|  | Naganawa S, Ishihara S, Iwano S, Kawai H., Sone M, Nakashima T. Estimation of gadolinium-induced T1-shortening with measurement of simple signal intensity ratio between the cochlea and brain parenchyma on 3D-FLAIR: Correlation with T1 measurement by TI scout sequence. Magn Reson Med Sci 2010; 9(1): 17-22. doi:10.2463/MRMS.9.17. | B |
|  | Naganawa S, Yamazaki M, Kawai H, Bokura K, Sone M, Nakashima T. Visualization of endolymphatic hydrops in Meniere’s disease with single-dose intravenous gadolinium-based contrast media using heavily T2-weighted 3D-FLAIR. Magn Reson Med Sci 2010; 9(4): 237-42. doi:10.2463/MRMS.9.237. | A |
|  | Naganawa S, Kawai H, Sone M, Nakashima T. Increased sensitivity to low concentration gadolinium contrast by optimized heavily T2weighted 3D-FLAIR to visualize endolymphatic space. Magn Reson Med Sci 2010; 9(2): 73-80. doi: 10.2463/mrms.9.73. | B |
|  | Naganawa S, Sone M, Yamazaki M, Kawai H., Nakashima T. Visualization of endolymphatic hydrops after intratympanic injection of GdDTPA: Comparison of 2D and 3D Real inversion recovery imaging. Magn Reson Med Sci 2011; 10(2):415-20. doi:  10.1097/MLG.0b013e31802c300c. | A |
|  | Naganawa S, Yamazaki M, Kawai H, Bokura K, Sone M, Nakashima T. Imaging of Meniere ’s disease after intravenous administration of single-dose gadodiamide: Utility of subtraction images with different inversion time. Magn Reson Med Sci 2012;11 (3):213-9. doi:  10.2463/mrms.11.213. | B |
|  | Naganawa S, Yamazaki M, Kawai H, Bokura K, Sone M, Nakashima T. Imaging of Meniere’s disease by subtraction of MR Cisternography from positive perilymph Image. Magn Reson Med Sci 2012; 11(4): 303-309. <https://doi.org/10.2463/mrms.11.303> | A |
|  | Naganawa S, Suzuki K, Nakamichi R et al. Semi-quantification of endolymphatic size on MR imaging after intravenous injection of single dose gadodiamide: Comparison between two types of processing strategies. Magn Reson Med Sci 2013; 12(4):261-269. doi:  10.2463/mrms.2013-0019. | C |
|  | Naganawa S, Yamazaki M, Kawai H, Bokura K, Sone M, Nakashima T. Estimation of perilymph enhancement after intratympanic administration of Gd-DTPA by fast T1-mapping with a dual flip angle 3D spoiled gradient echo sequence. Magn Reson Med Sci 2013; 12(3):  223-228. doi:10.2463/mrms.2012-0071 | A |
|  | Naganawa S, Yamazaki M, Kawai H, Bokura K, Sone M., Nakashima T. Imaging of Ménière’s disease after intravenous administration of single-dose gadodiamide: Utility of multiplication of MR cisternography and HYDROPS image. Magn Reson Med Sci 2013; 12(1), 63–68.  https://doi.org/10.2463/mrms.2012-0027 | A |
|  | Naganawa S, Yamazaki M, Kawai H, Bokura K, Sone M, Nakashima T. Visualization of endolymphatic hydrops in Méniere’s disease after intravenous administration of single-dose gadodiamide at 1.5T. Magn Reson Med Sci 2013; 12(2), 137–139.  <https://doi.org/10.2463/mrms.2012-0037> | A |
|  | Naganawa S, Kawai H, Ikeda M, Sone M, Nakashima T. Imaging of endolymphatic hydrops in 10 minutes: A new strategy to reduce scan time to one third. Magn Reson Med Sci 2015 14(1), 77–83. [https://doi.org/10.2463/mrms.2014-0065.](https://doi.org/10.2463/mrms.2014-0065) | A |
|  | Naganawa S, Ohashi T, Kanou M, Kuno K, Sone M, Ikeda M. Volume quantification of endolymph after intravenous administration of a single dose of gadolinium contrast agent: Comparison of 18- versus 8-minute imaging protocols. Magn Reson Med Sci 2015; 14(4): 257–  262. https://doi.org/10.2463/mrms.2014-0118 | B |
|  | Naganawa S, Kanou M, Ohashi T, Kuno K, Sone M. Simple estimation of the endolymphatic volume ratio after intravenous dministration of a single-dose of gadolinium contrast. Magnetic resonance in medical sciences: MRMS: an official journal of Japan Society of Magnetic  Resonance in Medicine 2016; 15:379-385. doi:10.2463/mrms.mp.2015-0175 | A |
|  | Naganawa S, Kawai H, Taoka T, Sone M. Improved HYDROPS: Imaging of Endolymphatic Hydrops after Intravenous Administration of  Gadolinium. Magnetic resonance in medical sciences: MRMS : an official journal of Japan Society of Magnetic Resonance in Medicine 2017;16(4):357-361. doi: [10.2463/mrms.tn.2016-0126](https://dx.doi.org/10.2463%2Fmrms.tn.2016-0126) | C |
|  | Naganawa S, Ito R, Kawai H, Kawamura M, Taoka T, Yoshida T, Sone M. Cross-sectional area of the superior petrosal sinus is reduced in patients with significant endolymphatic hydrops. Magn Reson Med Sci 2021; https://doi.org/10.2463/mrms.mp.2021-0010 | A |
|  | Naganawa S, Nakamichi R, Ichikawa K, Kawamura M, Kawai H, Yoshida T, Sone M. MR imaging of endolymphatic hydrops: Utility of iHYDROPS-Mi2 combined with deep learning reconstruction denoising. Magn Reson Med Sci 2021: 20(3): 272–279.  <https://doi.org/10.2463/mrms.mp.2020-0082> | A |
|  | Nakashima T, Naganawa S, Katayama N et al. Clinical significance of endolymphatic imaging after intratympanic gadolinium injection. Acta  Oto-Laryngologica 2009; 129(560): 9–14. https://doi.org/10.1080/00016480902729801 | A |
|  | Nakashima T, Naganawa S, Sugiura M et al. Visualization of endolymphatic hydrops in patients with Meniere’s disease. Laryngoscope  2007; 117(3): 415–420. <https://doi.org/10.1097/MLG.0b013e31802c300c> | A |
|  | Nakashima T, Naganawa S, Teranishi M et al. Endolymphatic hydrops revealed by intravenous gadolinium injection in patients with  Ménière’s disease. Acta Oto-Laryngologica 2010; 130(3): 338–343. <https://doi.org/10.3109/00016480903143986> | A |
|  | Neri G, Tartaro A, Neri L. MRI with intratympanic gadolinium: Comparison between otoneurological and radiological investigation in  Menière’s disease. Frontiers in Surgery 2021; 8. https://doi.org/10.3389/fsurg.2021.672284 | A |
|  | Nonoyama H, Tanigawa T, Tamaki T, Tanaka H, Yamamuro, O, Ueda H. Evidence for bilateral endolymphatic hydrops in ipsilateral delayed endolymphatic hydrops: Preliminary results from examination of five cases. Acta Oto-Laryngologica 2014; 134(3), 221–226.  https://doi.org/10.3109/00016489.2013.850741 | B |
|  | Ohashi T, Naganawa S, Takeuchi A, Katagir T, Kuno K. Quantification of endolymphatic space volume after intravenous administration of a single dose of gadolinium-based contrast agent: 3D-real inversion recovery versus HYDROPS-Mi2. Magn Reson Med Sci2020; 19(2), 119– 124. https://doi.org/10.2463/mrms.mp.2019-0013 | A |
|  | Okumura T, Imai T, Takimoto Y et al. Assessment of endolymphatic hydrops and otolith function in patients with Ménière’s disease.  European Archives of Oto-Rhino-Laryngology 2017; 274(3): 1413–1421. https://doi.org/10.1007/s00405-016-4418-2 | A |
|  | Osman S, Hautefort C, Attyé A , Vaussy, A , Houdart E, Eliezer M. Increased signal intensity with delayed post contrast 3D-FLAIR MRI sequence using constant flip angle and long repetition time for inner ear evaluation: Increased sensitivity to low concentration of gadolinium for inner ear MRI. Diagnostic and Interventional Imaging 2021. https://doi.org/10.1016/j.diii.2021.10.003 | B |
|  | Paškoniene A, Baltagalviene R, Lengvenis G et al. The Importance of the Temporal Bone 3T MR Imaging in the Diagnosis of Menière’s  Disease. Otology and Neurotology 2020; 41(2): 235–241. https://doi.org/10.1097/MAO.0000000000002471 | A |
|  | Peng A, Hu J, Wang, Q et al. Modulation of hearing function following the downgrading or upgrading of endolymphatic hydrops in  Meniere’s disease patients with endolymphatic duct blockage. PLoS ONE 2020; 15(10). https://doi.org/10.1371/journal.pone.0240315 | A |
|  | Sepahdari AR, Ishiyama G, Vorasubin N, Peng KA, Linetsky M, Ishiyama A. Delayed intravenous contrast-enhanced 3D FLAIR MRI in Meniere’s disease: Correlation of quantitative measures of endolymphatic hydrops with hearing. Clinical Imaging 2015; 39(1): 26–31.  https://doi.org/10.1016/j.clinimag.2014.09.014 | A |
|  | Sepahdari A, Vorasubin N, Ishiyama G., Ishiyama A. Endolymphatic hydrops reversal following acetazolamide therapy: Demonstration with delayed intravenous contrast-enhanced 3D-FLAIR MRI. American Journal of Neuroradiology 2016; 37(1): 151–154.  https://doi.org/10.3174/ajnr.A4462 | D |
|  | Shi S, Zhou F, Wang W. 3D-real IR MRI of Meniere's disease with partial endolymphatic hydrops. Am J Otol 2019; 40 (4): 589-593.  [https://doi.org/10.1016/j.amjoto.2019.05.015.](https://doi.org/10.1016/j.amjoto.2019.05.015) | D |
|  | Shi S, Zhou F, Wang W. 3D-real IR MRI of Meniere’s disease with partial endolymphatic hydrops. American Journal of Otolaryngology -  Head and Neck Medicine and Surgery 2019 40(4); 589–593. https://doi.org/10.1016/j.amjoto.2019.05.015 | A |
|  | Suga K, Kato M, Yoshida T et al. Changes in endolymphatic hydrops in patients with Ménière’s disease treated conservatively for more than 1 year. Acta Oto-Laryngologica 2015; 135(9): 866–870. https://doi.org/10.3109/00016489.2015.1015607 | A |
|  | Sugimoto S, Yoshida T, Teranishi M, Kobayashi M, Shimono M, Naganawa S, Sone M. Significance of endolymphatic hydrops herniation into the semicircular canals detected on MRI. Otol Neurotol 2018; 39(10), 1229–1234. https://doi.org/10.1097/MAO.0000000000002022 | D |
|  | Suzuki H, Teranishi M, Sone M, Yamazaki M, Naganawa S, Nakashima T. Contrast enhancement of the inner ear after intravenous administration of a standard or double dose of gadolinium contrast agents. Acta Oto-Laryngologica 2011; 131(10):1025-1031. | C |
|  | Suzuki H, Teranishi M, Naganawa S, Nakata S, Sone M., Nakashima T. Contrast-enhanced MRI of the inner ear after intratympanic injection of meglumine gadopentetate or gadodiamide hydrate. Acta Oto-Laryngologica 2011; 131(2): 130–135.  https://doi.org/10.3109/00016489.2010.507781 | A |
|  | Teranishi M, Naganawa S, Katayama N, Sugiura M, Nakata S, Sone M, Nakashima T. Image evaluation of endolymphatic space in fluctuating hearing loss without vertigo. European Archives of Oto-Rhino-Laryngology 2009; 266(12):1871–1877.  https://doi.org/10.1007/s00405-009-0989-5 | A |
|  | Tuñón Gómez M, Lobo Duro DR, Brea Álvarez B, García-Berrocal JR. Diagnosis of endolymphatic hydrops by means of 3 T magnetic resonance imaging after intratympanic administration of gadolinium. Radiología (English Edition) 2017; 59(2): 159–165.  https://doi.org/10.1016/j.rxeng.2017.02.001 | A |
|  | Uno A, Imai T, Watanabe Y et al. Changes in endolymphatic hydrops after sac surgery examined by Gd-enhanced MRI. Acta Oto-  Laryngologica 2013; 133(9), 924–929. https://doi.org/10.3109/00016489.2013.795290 | A |
|  | Wang J, Ren T, Sun W, Liang Q, Wang W. Post-contrast 3D-FLAIR in idiopathic sudden sensorineural hearing loss. Eur Arch  Otorhinolaryngol 2019; 276(5): 1291-1299. doi: [10.1007/s00405-019-05285-z](https://doi.org/10.1007/s00405-019-05285-z) | B |
|  | Wang P, Yu D, Wang H et al. Contrast-enhanced MRI combined with the glycerol test reveals the heterogeneous dynamics of endolymphatic hydrops in patients with Menière’s disease. Journal of Magnetic Resonance Imaging 2012; 52(4), 1066–1073.  https://doi.org/10.1002/jmri.27127 | A |
|  | Wu Q, Li X, Sha Y, Dai C. Clinical features and management of Meniere’s disease patients with drop attacks. European Archives of Oto-  Rhino-Laryngology 2019; 276(3), 665–672. https://doi.org/10.1007/s00405-018-5260-5 | A |
|  | Yamazaki M, Naganawa S, Kawai H, Nihashi T, Nakashima T. Signal alteration of the cochlear perilymph on 3 different sequences after intratympanic Gd-DTPA administration at 3 Tesla: Comparison of 3D-FLAIR, 3D-T1-weighted Imaging, and 3D-CISS. Magn Reson Med Sci 2010; 9(2):65-71. doi: 10.2463/mrms.9.65. | A |
|  | Yamazaki M, Naganawa S, Tagaya M et al. Comparison of contrast effect on the cochlear perilymph after intratympanic and intravenous gadolinium injection. American Journal of Neuroradiology 2012; 33(4), 773–778. https://doi.org/10.3174/ajnr.A2821. | A |
|  | Yamazaki M, Naganawa S, Kawai H, Sone M, Nakashima T. Gadolinium distribution in cochlear perilymph: Differences between intratympanic and intravenous gadolinium injection. Neuroradiology 2012; 54(10), 1161–1169. https://doi.org/10.1007/s00234-012-1078-  9 | A |
|  | Yoshioka M, Naganawa S, Sone M, Nakata S, Teranishi M, Nakashima T. Individual differences in the permeability of the round window:  Evaluating the movement of intratympanic gadolinium into the inner ear. Otol Neurotol 2009; 30: 645-648. doi: 10.1097/MAO.0b013e31819bda66 | B |
|  | Zhang Y, Cui Y hua, Hu Y. Changes in endolymphatic hydrops visualized by magnetic resonance imaging after sac surgery. Journal of  Huazhong University of Science and Technology - Medical Science 2016; 36(5): 736–740. https://doi.org/10.1007/s11596-016-1654-z | A |
|  | Zhang W, Hui L, Zhang B, Ren L, Zhu J, Wang F, Li S. The Correlation Between Endolymphatic Hydrops and Clinical Features of Meniere  Disease. Laryngoscope 2021; 131(1): e144–E150. https://doi.org/10.1002/lary.28576 | C |
|  | Zhang W, Xie J, Hui L, Li S, Zhang B. The Correlation Between Endolymphatic Hydrops and blood-labyrinth barrier Permeability of Meniere  Disease. Annals of Otology, Rhinology and Laryngology 2021; 130(6): 578–584. <https://doi.org/10.1177/0003489420964823> | A |
|  | Zheng Y, Liu A, Wang X long, Hu Y, Zhang Y, Peng L. The role of endolymphatic hydrops in patients with pantonal idiopathic sudden sensorineural hearing loss: A cause or secondary reaction. Current Medical Science 2019; 39(6): 972–977. https://doi.org/10.1007/s11596019-2130-3 | B |
|  | Zhu HL, Ou YK, Fu J, Zhang Y, Xiong H, Xu YD. A comparison of inner ear imaging features at different time points of sudden sensorineural hearing loss with three-dimensional fluid-attenuated inversion recovery magnetic resonance imaging. Eur Arch Otorhinolaryngol 2015; 272(10): 2659-65. doi: 10.1007/s00405-014-3187-z. | B |
|  | Zou J, Chen L, Li H, Zhang G, Pyykkö, I, Lu J. High-quality imaging of endolymphatic hydrops acquired in 7 minutes using sensitive hT2W–3D–FLAIR reconstructed with magnitude and zero-filled interpolation. European Archives of Oto-Rhino-Laryngology 2021; https://doi.org/10.1007/s00405-021-06912-4 | A |
