# Supplementary material for: The diagnostic performance of cochlear endolymphatic hydrops and perilymphatic enhancement in stratifying Ménière’s disease probabilities: A meta-analysis of semi-quantitative MRI-based grading systems
Source: PLoS One. 2024 Nov 21;19(11):e0310045. doi: 10.1371/journal.pone.0310045 (PMC11581247; doi:10.1371/journal.pone.0310045)
Supplement: S4 File — (DOCX) [file pone.0310045.s004.docx]

**Quality Assessment of Diagnostic Accuracy Studies-2 (QUADAS-2)**

**1. Patient Selection**

**Bias Questions:**

- **Q1:** Was the study sample genuinely consecutive or random, without post-hoc adjustments, to ensure the representativeness of the patient cohort with MD?
- **Q2:** Did the study avoid a case-control design, where cases of MD and controls are identified retrospectively, potentially leading to exaggerated estimates of diagnostic accuracy?
- **Q3:** Were all relevant patients included without exclusions based on criteria that could influence the likelihood of a positive or negative diagnostic outcome (e.g., excluding patients with atypical presentations or incomplete data)?

**Applicability Question:**

- **Q4:** Were the patients included in the study representative of those who would typically present in clinical practice with symptoms suggestive of Meniere’s disease and be considered for EH grading and PLE evaluation?

**2. Index Test**

**Bias Questions:**

- **Q5:** Were the results of the index tests (e.g., EH grading and PLE evaluation) interpreted strictly without any knowledge of the reference standard outcomes to prevent cognitive bias in interpreting imaging results?
- **Q6:** Were the thresholds or criteria for positive EH grading and PLE enhancement explicitly pre-defined and consistently applied across all subjects?

**Applicability Question:**

- **Q7:** Was the index test procedure, including the imaging modality and interpretation criteria, applied in a manner consistent with current clinical practice and guidelines for diagnosing Meniere’s disease?

**3. Reference Standard**

**Bias Questions:**

- **Q8:** Is the reference standard used in the study (e.g., clinical diagnosis of MD) validated and reliable enough to correctly classify the target condition without being influenced by the index test results?
- **Q9:** Were the results of the reference standard interpreted independently of the index test results to avoid any risk of bias from cross-influence?

**Applicability Question:**

- **Q10:** Is the reference standard used in the study appropriate for diagnosing Meniere’s disease in the context of the clinical settings to which the study results will be applied?

**4. Flow and Timing**

**Bias Questions:**

- **Q11:** Was the interval between the index test (EH grading/PLE evaluation) and the reference standard assessment short enough to avoid significant changes in the patient's condition that could alter the diagnostic outcomes?
- **Q12:** Did all patients in the study undergo the same reference standard after the index test, without variations that could introduce inconsistencies in the assessment of diagnostic performance?
- **Q13:** Were all patients included in the final analysis, with no exclusions of cases that might bias the results towards a more favorable outcome (e.g., excluding non-conclusive cases)?

**Considerations for QUADAS-2 Questions**

**Q2. Patient Selection Bias:**

In this context, all studies under evaluation inherently present a high risk of bias for patient selection. This is due to the consistent use of case-control designs where groups of diseased and control ears, often drawn from Meniere’s disease clinics or specific cohorts, are compared. Such designs tend to limit the diversity of the study population and may not adequately reflect the broader clinical variability seen in practice. This predisposes the results to spectrum bias, which is an inherent flaw in the design of these studies. Therefore, a high-risk assignment is uniformly justified for patient selection bias across all studies.

**Q4. Completeness of Clinical Diagnostic Criteria:**

For studies that comprehensively included the complete range of clinical diagnostic criteria for patients, a low risk of bias is assigned. This is because the use of exhaustive diagnostic criteria ensures that the study population is representative of the full spectrum of Meniere’s disease presentations. This reduces the likelihood of bias related to the underrepresentation or overrepresentation of certain patient subsets, thereby enhancing the study’s validity in assessing diagnostic accuracy.

**Q5. Conduct and Interpretation of the Test:**

A high risk of bias is attributed to the conduct and interpretation of the test in these studies. This arises primarily because the majority of studies focused exclusively on Meniere’s disease cohorts, and the blinding of observers was often not feasible. The lack of blinding introduces a significant risk of observer bias, as knowledge of the clinical context can influence the interpretation of diagnostic tests. This high-risk designation reflects the inherent challenges in achieving unbiased test interpretation in this specific research context.

**Q6. Predefined Grading Systems:**

Given that only studies utilizing predefined grading systems were included in the analysis, a low risk of bias is consistently assigned for this question. The use of standardized grading systems minimizes the potential for subjective interpretation and enhances the reliability of the diagnostic assessments across different studies.

**Q8. Reference Standard Bias:**

A high risk of bias is uniformly assigned in the reference standard domain due to the absence of a definitive gold standard for diagnosing Meniere’s disease. Without a universally accepted or clearly superior diagnostic reference, there is significant uncertainty regarding the accuracy of the diagnostic evaluations. This lack of a robust reference standard complicates the interpretation of results, necessitating a high-risk assignment for this domain.

**Q10. Applicability of Clinical Assessments:**

Low concern is attributed to the applicability of clinical assessments (Q10) across all studies in this analysis. This low concern stems from the consistent use of clinically verified assessment protocols, as it was part of the inclusion criteria. These established guidelines ensure that the diagnostic criteria and processes are relevant and generalizable to clinical practice.

**Table. QUADAS-2 Assessment**

| Reference | Q1: Consecutive/Random Sampling | Q2: Case-Control Design Avoidance | Q3: Inclusion of All Relevant Patients | Q4: Representativeness of Study Population | Q5: Blinding of Index Test Interpretation | Q6: Pre-Defined Criteria for Index Test | Q7: Applicability of Index Test Procedure | Q8: Validated Reference Standard | Q9: Independent Interpretation of Reference Standard | Q10: Applicability of Reference Standard | Q11: Timing Between Index Test and Reference Standard | Q12: Uniform Application of Reference Standard | Q13: Inclusion of All Patients in Analysis |
| --- | --- | --- | --- | --- | --- | --- | --- | --- | --- | --- | --- | --- | --- |
| Bernaerts 2019[1] | **?** | **✗** | **✓** | **✓** | **✗** | **✓** | **✓** | **✗** | **?** | **✓** | **?** | **✓** | **✓** |
| Bernaerts 2022[2] | **✓** | **✗** | **✓** | **✓** | **✗** | **✓** | **✓** | **✗** | **?** | **✓** | **✓** | **✓** | **✓** |
| Chen 2021[3] | **✓** | **✗** | **✓** | **✓** | **✗** | **✓** | **✓** | **✗** | **✓** | **✓** | **?** | **✓** | **✓** |
| Connor 2022[4] | **?** | **✗** | **✓** | **✓** | **✗** | **✓** | **✓** | **✗** | **?** | **✓** | **✓** | **✓** | **✓** |
| Conte 2018[5] | **?** | **✗** | **✓** | **✓** | **✗** | **✓** | **✓** | **✗** | **?** | **✓** | **?** | **?** | **✓** |
| Domínguez 2021[6] | **?** | **✗** | **?** | **✓** | **✓** | **✓** | **✓** | **✗** | **?** | **✓** | **?** | **✓** | **?** |
| Guajardo-Vergara 2022[7] | **✓** | **✗** | **✓** | **✓** | **✗** | **✓** | **✓** | **✗** | **?** | **✓** | **✓** | **✓** | **✓** |
| Han 2022[8] | **?** | **✗** | **✓** | **✓** | **✗** | **✓** | **✓** | **✗** | **✓** | **✓** | **✓** | **✓** | **✓** |
| Jasinska 2022[9] | **?** | **✗** | **✓** | **✓** | **✗** | **✓** | **✓** | **✗** | **?** | **✓** | **✓** | **✓** | **✓** |
| Kahn 2019[10] | **?** | **✗** | **✓** | **✓** | **✗** | **✓** | **✓** | **✗** | **✓** | **✓** | **✓** | **✓** | **✓** |
| Kazemi 2022[11] | **?** | **✗** | **✓** | **✓** | **✗** | **✓** | **✓** | **✗** | **?** | **✓** | **?** | **✓** | **✓** |
| Kenis 2021[12] | **?** | **✗** | **✓** | **✓** | **✗** | **✓** | **✓** | **✗** | **?** | **✓** | **?** | **✓** | **✓** |
| Kirbac 2022[13] | **?** | **✗** | **✓** | **✓** | **✗** | **✓** | **✓** | **✗** | **?** | **✓** | **✓** | **✓** | **✓** |
| Li 2020[14] | **?** | **✗** | **✓** | **✓** | **✗** | **✓** | **✓** | **✗** | **?** | **✓** | **?** | **✓** | **✓** |
| Mainnemarre 2020[15] | **?** | **✗** | **✓** | **✓** | **✗** | **✓** | **✓** | **✗** | **?** | **✓** | **?** | **✓** | **✓** |
| Morimoto 2017[16] | **?** | **✗** | **✓** | **✓** | **✗** | **✓** | **✓** | **✗** | **?** | **✓** | **?** | **✓** | **✓** |
| Morimoto 2020[17] | **?** | **✗** | **✓** | **✓** | **✗** | **✓** | **✓** | **✗** | **?** | **✓** | **?** | **✓** | **✓** |
| Morita 2020[18] | **?** | **✗** | **✓** | **✓** | **✗** | **✓** | **✓** | **✗** | **?** | **✓** | **?** | **✓** | **✓** |
| Naganawa 2014[19] | **?** | **✗** | **✓** | **?** | **✗** | **✓** | **✓** | **✗** | **?** | **✓** | **?** | **?** | **✓** |
| Nahmani 2020[20] | **?** | **✗** | **✓** | **✓** | **✗** | **✓** | **✓** | **✗** | **?** | **✓** | **?** | **✓** | **✓** |
| Oh 2021[21] | **?** | **✗** | **✓** | **✓** | **✗** | **✓** | **✓** | **✗** | **?** | **✓** | **?** | **✓** | **✓** |
| Okazaki 2017[22] | **?** | **✗** | **✓** | **✓** | **✗** | **✓** | **✓** | **✗** | **?** | **✓** | **?** | **?** | **✓** |
| Pai 2020[23] | **?** | **✗** | **✓** | **✓** | **✗** | **✓** | **✓** | **✗** | **?** | **✓** | **?** | **✓** | **✓** |
| Pyykkö 2013[24] | **?** | **✗** | **✓** | **✓** | **✗** | **✓** | **✓** | **✗** | **?** | **✓** | **?** | **✓** | **✓** |
| Sano 2012[25] | **?** | **✗** | **✓** | **✓** | **✗** | **✓** | **✓** | **✗** | **?** | **✓** | **?** | **✓** | **✓** |
| Shi 2018[26] | **?** | **✗** | **✓** | **✓** | **✗** | **✓** | **✓** | **✗** | **✓** | **✓** | **?** | **✓** | **✓** |
| Shiraishi 2020[27] | **?** | **✗** | **✓** | **✓** | **✗** | **✓** | **✓** | **✗** | **✓** | **✓** | **?** | **✓** | **✓** |
| Sousa 2022[28] | **?** | **✗** | **✓** | **✓** | **✗** | **✓** | **✓** | **✗** | **✓** | **✓** | **?** | **✓** | **✓** |
| Suárez Vega 2020[29] | **✓** | **✗** | **✓** | **✓** | **✗** | **✓** | **✓** | **✗** | **✓** | **✓** | **?** | **✓** | **✓** |
| Tagaya 2011[30] | **?** | **✗** | **?** | **✓** | **✗** | **✓** | **✓** | **✗** | **?** | **✓** | **?** | **✓** | **✓** |
| van Steekelenburg 2020[31] | **?** | **✗** | **?** | **✓** | **✗** | **✓** | **✓** | **✗** | **?** | **✓** | **?** | **✓** | **✓** |
| Wu 2016[32] | **?** | **✗** | **?** | **✓** | **✗** | **✓** | **✓** | **✗** | **✓** | **✓** | **?** | **✓** | **✓** |
| Xie 2021[33] | **?** | **✗** | **?** | **✓** | **✗** | **✓** | **✓** | **✗** | **✓** | **✓** | **?** | **✓** | **✓** |
| Yamamoto 2010[34] | **?** | **✗** | **?** | **✓** | **✗** | **✓** | **✓** | **✗** | **✓** | **✓** | **✓** | **✓** | **✓** |
| Yoshida 2018[35] | **?** | **✗** | **?** | **✓** | **✗** | **✓** | **✓** | **✗** | **✓** | **✓** | **✓** | **✓** | **✓** |

**^✓^ Low Risk:** Minimal potential for bias, with robust and reliable methodological practices.

**^✗^ High Risk:** Significant potential for bias or methodological concerns that may compromise validity.

**^?^ Unclear Risk:** Indeterminate level of risk due to insufficient or ambiguous information.

1. Bernaerts, A.; Vanspauwen, R.; Blaivie, C.; van Dinther, J.; Zarowski, A.; Wuyts, F.L.; Vanden Bossche, S.; Offeciers, E.; Casselman, J.W.; De Foer, B. The value of four stage vestibular hydrops grading and asymmetric perilymphatic enhancement in the diagnosis of Menière's disease on MRI. *Neuroradiology* **2019**, *61*, 421-429, doi:10.1007/s00234-019-02155-7.

2. Bernaerts, A.; Janssen, N.; Wuyts, F.L.; Blaivie, C.; Vanspauwen, R.; van Dinther, J.; Zarowski, A.; Offeciers, E.; Deckers, F.; Casselman, J.W.; et al. Comparison between 3D SPACE FLAIR and 3D TSE FLAIR in Menière's disease. *Neuroradiology* **2022**, *64*, 1011-1020, doi:10.1007/s00234-022-02913-0.

3. Chen, W.; Geng, Y.; Lin, N.; Yu, S.; Sha, Y. Magnetic resonance imaging with intravenous gadoteridol injection based on 3D-real IR sequence of the inner ear in Meniere's disease patient: feasibility in 3.5-h time interval. *Acta Otolaryngol* **2021**, *141*, 899-906, doi:10.1080/00016489.2021.1973681.

4. Pai, I.; Connor, S. Low Frequency Air-Bone Gap in Meniere's Disease: Relationship With Magnetic Resonance Imaging Features of Endolymphatic Hydrops. *Ear Hear* **2022**, *43*, 1678-1686, doi:10.1097/aud.0000000000001231.

5. Conte, G.; Caschera, L.; Calloni, S.; Barozzi, S.; Di Berardino, F.; Zanetti, D.; Scuffi, C.; Scola, E.; Sina, C.; Triulzi, F. MR Imaging in Menière Disease: Is the Contact between the Vestibular Endolymphatic Space and the Oval Window a Reliable Biomarker? *AJNR Am J Neuroradiol* **2018**, *39*, 2114-2119, doi:10.3174/ajnr.A5841.

6. Domínguez, P.; Manrique-Huarte, R.; Suárez-Vega, V.; López-Laguna, N.; Guajardo, C.; Pérez-Fernández, N. Endolymphatic Hydrops in Fluctuating Hearing Loss and Recurrent Vertigo. *Front Surg* **2021**, *8*, 673847, doi:10.3389/fsurg.2021.673847.

7. Guajardo-Vergara, C.; Suárez-Vega, V.; Dominguez, P.; Manrique-Huarte, R.; Arbizu, L.; Pérez-Fernández, N. Endolymphatic hydrops in the unaffected ear of patients with unilateral Ménière's disease. *Eur Arch Otorhinolaryngol* **2022**, *279*, 5591-5600, doi:10.1007/s00405-022-07412-9.

8. Han, S.C.; Kim, Y.S.; Kim, Y.; Lee, S.Y.; Song, J.J.; Choi, B.Y.; Kim, J.S.; Bae, Y.J.; Koo, J.W. Correlation of clinical parameters with endolymphatic hydrops on MRI in Meniere's disease. *Front Neurol* **2022**, *13*, 937703, doi:10.3389/fneur.2022.937703.

9. Jasińska, A.; Lachowska, M.; Wnuk, E.; Pierchała, K.; Rowiński, O.; Niemczyk, K. Correlation between magnetic resonance imaging classification of endolymphatic hydrops and clinical manifestations and audiovestibular test results in patients with definite Ménière's disease. *Auris Nasus Larynx* **2022**, *49*, 34-45, doi:10.1016/j.anl.2021.03.027.

10. Kahn, L.; Hautefort, C.; Guichard, J.P.; Toupet, M.; Jourdaine, C.; Vitaux, H.; Herman, P.; Kania, R.; Houdart, E.; Attyé, A.; et al. Relationship between video head impulse test, ocular and cervical vestibular evoked myogenic potentials, and compartmental magnetic resonance imaging classification in menière's disease. *Laryngoscope* **2020**, *130*, E444-e452, doi:10.1002/lary.28362.

11. Kazemi, M.A.; Ghasemi, A.; Casselman, J.W.; Shafiei, M.; Zarandy, M.M.; Sharifian, H.; Hashemi, H.; Firouznia, K.; Moradi, B.; Kasani, K.; et al. Correlation of semi-quantitative findings of endolymphatic hydrops in MRI with the audiometric findings in patients with Meniere's disease. *J Otol* **2022**, *17*, 123-129, doi:10.1016/j.joto.2022.04.001.

12. Kenis, C.; Crins, T.; Bernaerts, A.; Casselman, J.; Foer, B. Diagnosis of Menière's disease on MRI: feasibility at 1.5 Tesla. *Acta Radiol* **2022**, *63*, 810-813, doi:10.1177/02841851211016478.

13. Kirbac, A.; Incesulu, S.A.; Toprak, U.; Caklı, H.; Ozen, H.; Saylisoy, S. Audio-vestibular and radiological analysis in Meniere's disease. *Braz J Otorhinolaryngol* **2022**, *88 Suppl 3*, S117-s124, doi:10.1016/j.bjorl.2022.08.003.

14. Li, X.; Wu, Q.; Sha, Y.; Dai, C.; Zhang, R. Gadolinium-enhanced MRI reveals dynamic development of endolymphatic hydrops in Ménière's disease. *Braz J Otorhinolaryngol* **2020**, *86*, 165-173, doi:10.1016/j.bjorl.2018.10.014.

15. Mainnemarre, J.; Hautefort, C.; Toupet, M.; Guichard, J.P.; Houdart, E.; Attyé, A.; Eliezer, M. The vestibular aqueduct ossification on temporal bone CT: an old sign revisited to rule out the presence of endolymphatic hydrops in Menière's disease patients. *Eur Radiol* **2020**, *30*, 6331-6338, doi:10.1007/s00330-020-06980-w.

16. Morimoto, K.; Yoshida, T.; Sugiura, S.; Kato, M.; Kato, K.; Teranishi, M.; Naganawa, S.; Nakashima, T.; Sone, M. Endolymphatic hydrops in patients with unilateral and bilateral Meniere's disease. *Acta Otolaryngol* **2017**, *137*, 23-28, doi:10.1080/00016489.2016.1217042.

17. Morimoto, K.; Yoshida, T.; Kobayashi, M.; Sugimoto, S.; Nishio, N.; Teranishi, M.; Naganawa, S.; Sone, M. Significance of high signal intensity in the endolymphatic duct on magnetic resonance imaging in ears with otological disorders. *Acta Otolaryngol* **2020**, *140*, 818-822, doi:10.1080/00016489.2020.1781927.

18. Morita, Y.; Takahashi, K.; Ohshima, S.; Yagi, C.; Kitazawa, M.; Yamagishi, T.; Izumi, S.; Horii, A. Is Vestibular Meniere's Disease Associated With Endolymphatic Hydrops? *Front Surg* **2020**, *7*, 601692, doi:10.3389/fsurg.2020.601692.

19. Naganawa, S.; Yamazaki, M.; Kawai, H.; Bokura, K.; Iida, T.; Sone, M.; Nakashima, T. MR imaging of Ménière's disease after combined intratympanic and intravenous injection of gadolinium using HYDROPS2. *Magn Reson Med Sci* **2014**, *13*, 133-137, doi:10.2463/mrms.2013-0061.

20. Nahmani, S.; Vaussy, A.; Hautefort, C.; Guichard, J.P.; Guillonet, A.; Houdart, E.; Attyé, A.; Eliezer, M. Comparison of Enhancement of the Vestibular Perilymph between Variable and Constant Flip Angle-Delayed 3D-FLAIR Sequences in Menière Disease. *AJNR Am J Neuroradiol* **2020**, *41*, 706-711, doi:10.3174/ajnr.A6483.

21. Oh, S.Y.; Dieterich, M.; Lee, B.N.; Boegle, R.; Kang, J.J.; Lee, N.R.; Gerb, J.; Hwang, S.B.; Kirsch, V. Endolymphatic Hydrops in Patients With Vestibular Migraine and Concurrent Meniere's Disease. *Front Neurol* **2021**, *12*, 594481, doi:10.3389/fneur.2021.594481.

22. Okazaki, Y.; Yoshida, T.; Sugimoto, S.; Teranishi, M.; Kato, K.; Naganawa, S.; Sone, M. Significance of Endolymphatic Hydrops in Ears With Unilateral Sensorineural Hearing Loss. *Otol Neurotol* **2017**, *38*, 1076-1080, doi:10.1097/mao.0000000000001499.

23. Pai, I.; Mendis, S.; Murdin, L.; Touska, P.; Connor, S. Magnetic resonance imaging of Ménière's disease: early clinical experience in a UK centre. *J Laryngol Otol* **2020**, *134*, 302-310, doi:10.1017/s0022215120000626.

24. Pyykkö, I.; Nakashima, T.; Yoshida, T.; Zou, J.; Naganawa, S. Meniere's disease: a reappraisal supported by a variable latency of symptoms and the MRI visualisation of endolymphatic hydrops. *BMJ Open* **2013**, *3*, doi:10.1136/bmjopen-2012-001555.

25. Sano, R.; Teranishi, M.; Yamazaki, M.; Isoda, H.; Naganawa, S.; Sone, M.; Hiramatsu, M.; Yoshida, T.; Suzuki, H.; Nakashima, T. Contrast enhancement of the inner ear in magnetic resonance images taken at 10 minutes or 4 hours after intravenous gadolinium injection. *Acta Otolaryngol* **2012**, *132*, 241-246, doi:10.3109/00016489.2011.639085.

26. Shi, S.; Guo, P.; Wang, W. Magnetic Resonance Imaging of Ménière's Disease After Intravenous Administration of Gadolinium. *Ann Otol Rhinol Laryngol* **2018**, *127*, 777-782, doi:10.1177/0003489418794699.

27. Shiraishi, K.; Ohira, N.; Kobayashi, T.; Sato, M.; Osaki, Y.; Doi, K. Comparison of furosemide-loading cervical vestibular-evoked myogenic potentials with magnetic resonance imaging for the evaluation of endolymphatic hydrops. *Acta Otolaryngol* **2020**, *140*, 723-727, doi:10.1080/00016489.2020.1769863.

28. Sousa, R.; Lobo, M.; Cadilha, H.; Eça, T.; Campos, J.; Luis, L. Is there progression of endolymphatic hydrops in Ménière's disease? Longitudinal magnetic resonance study. *Eur Arch Otorhinolaryngol* **2022**, doi:10.1007/s00405-022-07721-z.

29. Suárez Vega, V.M.; Dominguez, P.; Caballeros Lam, F.M.; Leal, J.I.; Perez-Fernandez, N. Comparison between high-resolution 3D-IR with real reconstruction and 3D-flair sequences in the assessment of endolymphatic hydrops in 3 tesla. *Acta Otolaryngol* **2020**, *140*, 883-888, doi:10.1080/00016489.2020.1792550.

30. Tagaya, M.; Yamazaki, M.; Teranishi, M.; Naganawa, S.; Yoshida, T.; Otake, H.; Nakata, S.; Sone, M.; Nakashima, T. Endolymphatic hydrops and blood-labyrinth barrier in Ménière's disease. *Acta Otolaryngol* **2011**, *131*, 474-479, doi:10.3109/00016489.2010.534114.

31. van Steekelenburg, J.M.; van Weijnen, A.; de Pont, L.M.H.; Vijlbrief, O.D.; Bommeljé, C.C.; Koopman, J.P.; Verbist, B.M.; Blom, H.M.; Hammer, S. Value of Endolymphatic Hydrops and Perilymph Signal Intensity in Suspected Ménière Disease. *AJNR Am J Neuroradiol* **2020**, *41*, 529-534, doi:10.3174/ajnr.A6410.

32. Wu, Q.; Dai, C.; Zhao, M.; Sha, Y. The correlation between symptoms of definite Meniere's disease and endolymphatic hydrops visualized by magnetic resonance imaging. *Laryngoscope* **2016**, *126*, 974-979, doi:10.1002/lary.25576.

33. Xie, J.; Zhang, W.; Zhu, J.; Hui, L.; Li, S.; Ren, L.; Wang, F.; Zhang, B. Differential Diagnosis of Endolymphatic Hydrops Between "Probable" and "Definite" Ménière's Disease via Magnetic Resonance Imaging. *Otolaryngol Head Neck Surg* **2021**, *165*, 696-700, doi:10.1177/0194599821990680.

34. Yamamoto, M.; Teranishi, M.; Naganawa, S.; Otake, H.; Sugiura, M.; Iwata, T.; Yoshida, T.; Katayama, N.; Nakata, S.; Sone, M.; et al. Relationship between the degree of endolymphatic hydrops and electrocochleography. *Audiol Neurootol* **2010**, *15*, 254-260, doi:10.1159/000258681.

35. Yoshida, T.; Sugimoto, S.; Teranishi, M.; Otake, H.; Yamazaki, M.; Naganawa, S.; Nakashima, T.; Sone, M. Imaging of the endolymphatic space in patients with Ménière's disease. *Auris Nasus Larynx* **2018**, *45*, 33-38, doi:10.1016/j.anl.2017.02.002.
